# Protamine cleavage specificity of the avian pathogen *Escherichia coli* OmpT reveals two substrate-binding sites related to virulence

**DOI:** 10.3389/fvets.2024.1410113

**Published:** 2024-09-05

**Authors:** Juanhua Liu, Luyao Jiang, Hang Wang, Jiayan Wu, Qingqing Gao, Changchao Huan, Song Gao

**Affiliations:** ^1^Jiangsu Co-Innovation Center for Prevention and Control of Important Animal Infectious Diseases and Zoonoses, College of Veterinary Medicine, Yangzhou University, Yangzhou, China; ^2^The International Joint Laboratory for Cooperation in Agriculture and Agricultural Product Safety, Ministry of Education, Yangzhou, China

**Keywords:** avian pathogenic *Escherichia coli*, proteolytic activity, cleavage specificity, substrate affinity, substrate-binding site, virulence

## Abstract

The pathogenic nature of bacteria can be increased by cleaving antimicrobial peptides using omptins, to avoid or counter the host’s natural immune defenses. Plasmid-encoded OmpT (pOmpT or ArlC) in avian pathogenic *Escherichia coli* (APEC), like the chromosome-encoded OmpT (cOmpT), belongs to the omptin family and both exhibit highly similar sequences and structures. Through sequence alignment and physiological examinations, pOmpT has been identified as a virulence factor, distinct from cOmpT in terms of substrate specificity. When pOmpT is compared with cOmpT regarding their proteolytic activities and target substrates, Asp^267^ and Ser^276^ on loop 5 of cOmpT are found to be binding sites that facilitate substrate anchoring and enhance substrate cleavage (protamine or synthetic peptide) by the catalytic center. Conversely, the characteristics of residues at positions 267 and 276 on loop 5 of pOmpT inhibit protamine cleavage, yet allow the specific cleavage of the human antimicrobial peptide RNase 7, which plays a role in host defense. This finding suggests a relationship between these two binding sites and substrate specificity. Furthermore, the substrate-binding sites (residues 267 and 276, particularly residue 267) of cOmpT and pOmpT are determined to be critical in the virulence of APEC. In summary, residues 267 and 276 of pOmpT are crucial for the pathogenicity of APEC and offer new insights into the determinants of APEC virulence and the development of antimicrobial drugs.

## 1 Introduction

*Escherichia coli* (*E. coli*) is a common bacterium found in the gastrointestinal tract of mammals and birds, but it can also cause a range of severe diseases in humans and animals due to its multifunctional pathogenic nature. In poultry, avian pathogenic *E. coli* (APEC), an extraintestinal pathogenic *E. coli* (ExPEC), acting either as a primary or secondary agent can infect all types of birds at all ages, causing localized and systemic infections, often referred to as avian colibacillosis. At present, avian colibacillosis is one of the leading causes of mortality and morbidity associated with economic losses in the poultry industry throughout the world. The development of bacterial antibiotic resistance and the ineffectiveness of vaccines pose significant risks to public health and the poultry industry. Consequently, there is an urgent need for more effective prevention and treatment strategies.

Antimicrobial peptides (AMPs) represent one of the primary challenges that bacterial pathogens encounter when infecting a host, playing a crucial role in the innate immune system (1). These peptides, small in size (20–50 amino acids), cationic, and amphiphilic, are primarily secreted by the host’s epithelial cells and neutrophils (2–4). Their ability to attach to anionic bacterial membranes, create pores, and consequently cause bacterial death through cell lysis is well-documented (5–7). Beyond their bactericidal capabilities, AMPs also have the capacity to attract immune cells of the host to infection sites, thereby inducing a wide spectrum of immunomodulatory activities to manage bacterial infections (8).

Despite AMPs’ critical role in the host’s defense mechanism, bacteria have developed numerous strategies to counteract the effects of AMPs, such as utilizing LPS modifications, efflux pumps, capsules, and proteases (1). Omptins, a distinct group of outer membrane (OM) proteases with proteolytic capabilities, have garnered significant attention. Found across various Gram-negative bacteria within the Enterobacteriaceae family, these proteases influence bacterial virulence through the modification or degradation of numerous host and bacterial proteins (9–19). Currently identified omptins include OmpT, OmpP, and ArlC in *E. coli*, Pla in *Yersinia pestis*, PgtE in *Salmonella enteritidis*, IcsP in *Shigella flexneri*, and CroP in *Citrobacter rodentium*. They share a high degree of amino acid sequence similarity (45–80%) with highly conserved active sites (11, 19–24). OmpT, the first to be characterized and encoded by the *E. coli* chromosome, is a 37 kDa protein that forms a hollow β-barrel structure with its active sites exposed to the external environment (24, 25). Inhibitory studies on omptin have shown that elements such as Zn^2+^, Cu^2+^, and benzamidine can hinder OmpT activity (26–28), while the serine protease inhibitors aprotinin and ulinastatin can also disrupt OmpT’s functionality (29, 30). Additionally, the highly conserved structure of OmpT and the nature of its active site residues (residues Asp^83^, Asp^85^, Asp^210^, and His^212^) are crucial for its substrate cleavage specificity, often preferring dibasic motifs (24, 31–33). It has been observed that OmpT in urinary pathogenic *E. coli* (UPEC) aids in bacterial survival within the host by cleaving secreted antimicrobial peptides like protamine P1 and cathelicidin LL-37 from human urethral epithelial cells (13, 34–36). Furthermore, OmpT present in enterohemorrhagic *E. coli* (EHEC) and enteropathogenic *E. coli* (EPEC) can cleave and inactivate LL-37 to varying degrees (12, 37). The plasmid-encoded OmpT-like protease OmpP, found in isolates from urinary tract infections (UTIs), has been shown to cleave the AMP protamine, and ArlC is linked to AMP resistance (e.g., human AMP RNase 7); thus, aiding bacterial survival (23, 38, 39).

Omptins, as key virulence factors that play a core role in the host-interface, are potential targets for antimicrobial and vaccine development, so they have research potential. In fact, for most *E. coli*, OmpT is only encoded by the *ompT* gene on the chromosome. Previous studies have focused mainly on the proteolytic activity and pathogenicity of OmpT located on the chromosome (24, 25, 32, 40). We found that the avian pathogenic *E. coli* (APEC) E058 strain has both the OmpT (cOmpT) gene (c*ompT*) on its chromosome and the OmpT (pOmpT) gene encoded by the *ColV* plasmid (p*ompT*), both of which exhibit highly homologous amino acid sequences up to about 76 percent and are involved in the pathogenicity of APEC in the host (40, 41). Although cOmpT has been extensively studied, research on pOmpT is relatively scarce. Here, we aimed to explore the special role of pOmpT in physiology compared with that of cOmpT and to clarify the effect of pOmpT on APEC pathogenicity both *in vitro* and *in vivo*. The in-depth study of proteases such as OmpT is conducive to designing new enzyme-resistant antimicrobial peptides and provides a possibility for the exploration of new anti-infection approaches and the screening of new drug targets.

## 2 Materials and methods

### 2.1 Bacterial strains, plasmids, antibodies, and growth conditions

The bacterial strains and plasmids used in this study are listed in Supplementary Table S1. The oligonucleotide primers used are listed in Supplementary Table S2. The APEC strain E058 was isolated from a chicken with the typical clinical symptoms of colibacillosis in China (42). OmpT mouse-origin monoclonal antibody was developed by our laboratory (43). The other details related to the strains are as follows. The strains were grown in Luria–Bertani (LB) broth, on LB agar plates or in N-minimal medium (44) adjusted to pH 7.5 and supplemented with 0.2% glucose and 1 mM MgCl_2_. Antibiotics such as 50 μg/mL kanamycin, 50 μg/mL spectinomycin, 30 μg/mL chloramphenicol or 50 μg/mL tetracycline were used for selection wherever needed. All the cultures were grown at 37°C or 30°C under aerobic conditions.

### 2.2 Generation of mutants, revertants, and recombinant expression bacteria

The bacterial strains used in this study are listed in Supplementary Table S1. Single/double gene deletions of the c*ompT* and p*ompT* genes of the APEC E058 strain were performed using the lambda red recombinase system (41). Simultaneously, the native c*ompT* and p*ompT* genes, alongside their putative promoters, were amplified, cloned, and inserted into the plasmid pACYC184. Following PCR and DNA sequencing, the plasmids p184-cc*ompT* and p184-pp*ompT* were transformed into the c*ompT*/p*ompT* single gene deletion strains to create complementation strains through electroporation.

To minimize interference from outer membrane proteins other than the cOmpT/pOmpT protein in the E058 strain, the c*ompT* and p*ompT* genes’ entire open reading frame (ORF) was cloned and inserted into the expression plasmid pET-28a. Subsequently, the correct plasmids were transformed into *E. coli* BL21(DE3), which naturally lacks the *ompT* gene, after PCR and DNA sequencing. Furthermore, the amino acid sequences of the cOmpT and pOmpT proteins were divided into five loops based on the topological structure of the cOmpT protein. Employing fusion PCR technology, we utilized the same gene cloning method as mentioned earlier to construct a series of recombinant bacteria with corresponding loop interchanges and site-directed mutations in loop 5 (L5). To further determine the importance of L5 and the amino acid at positions 83/85/210/212/267/276 of cOmpT/pOmpT in the wild-type strain E058, mutual interference between cOmpT and pOmpT must be excluded. Therefore, we first inserted chimeric or site-directed mutant DNA fragments via gene deletion and then deleted or inserted another gene on the basis of the c*ompT*/p*ompT* single-gene deletion strain using a CRISPR-Cas9 system-based continual genome editing strategy (45). The guide sequence (N20 sequence), which targets the FRT sequence of the c*ompT*/p*ompT* single-gene mutant strain and the c*ompT* and p*ompT* gene sequences, was used to construct pTarget series plasmids. The donor DNA was amplified correspondingly using the genomic DNA of the c*ompT*/p*ompT* single-gene deletion strain and the above correct pET series plasmids with 5 loops interchange and sites mutation/interchange of the c*ompT*/p*ompT* gene as templates.

The DNA template of RNase 7 was derived from the experimenter’s nasal swab sample. The *rnase 7* gene without a signal peptide and with a His tag and site-directed mutations was amplified by overlap PCR and subsequently cloned and inserted into the expression plasmid pDEST17 after digestion. Then, the correct plasmid pDEST17-RNase 7 was transformed into competent BL21(AI) cells (46, 47).

### 2.3 Bacterial RNA isolation, RT–PCR/qPCR, sequencing, and alignment analysis

Total RNA was extracted from the APEC strain E058 and reverse-transcribed into cDNA using the PrimeScript RT reagent kit (TaKaRa, China) according to the manufacturer’s protocol. Primer sets for the PCR amplification of the target genes c*ompT* and p*ompT* in cDNA samples are detailed in Supplementary Table S2. Concurrently, PCRs were conducted using strain E058 DNA as positive controls and cDNA samples without reverse transcription (RT) activation as negative controls. The PCR products were separated on 0.8% agarose gels. Then, the fragments corresponding to the PCR-amplified genes c*ompT* and p*ompT* were extracted from the agarose gels using an Axygen DNA gel extraction kit (Corning, China) and were subjected to sequencing verification. The qPCR system was followed with ChamQ SYBR qPCR Master Mix (Vazyme, China). The thermal conditions were as follows: 95°C for 30 s, 40 cycles of 95°C for 10 s, and 60°C for 30 s. The *gapA* gene was used as the internal control for normalization. The primers used for qRT–PCR of the c*ompT* and p*ompT* genes are listed in Supplementary Table S2. Sequence alignment was performed among the amino acid sequences of the c*ompT* and p*ompT* genes of APEC E058, the *ompT* gene amino acid sequence located on the chromosome of the *E. coli* strain K12 substrain MG1655 (NC_000913.3), UPEC CFT073 (CP051263.1), AIEC NRG857C (CP001855.1) and UPEC isolate cystitis 6 (CP041302.1) and the amino acid sequence of the *arlC* gene located on the AIEC NRG857C (CP001856.1) and UPEC isolate cystitis 6 (CP041301.1) plasmids published in the NCBI.

### 2.4 Outer membrane protein extraction

Bacteria were cultured overnight in 200 mL N-minimal medium. The isolation of outer membrane fractions was performed as follows (48): bacterial cells were centrifuged at 6,000 rpm for 10 min at 4°C, and the pellets were resuspended in 7 mL HEPES buffer (10 mM HEPES, pH 7.4) and sonicated. A 1 mL aliquot of the lysate was reserved as whole bacterial protein for subsequent use. The remaining samples were then centrifuged at 6,000 rpm for 10 min at 4°C. The supernatants were collected and supplemented with 48 mL of sarcosyl buffer (2% sarcosyl), followed by a 30-min incubation at 4°C. After centrifugation for 1 h at 35,000 rpm, the pellet containing the outer membrane protein was resuspended in 1 mL of buffer A (20 mM Tris–HCl pH 7.5, 10% glycerol) for later analysis.

### 2.5 Western blotting

A BCA protein concentration determination kit (Beyotime, China) was used to determine the concentration of the total bacterial protein and total outer membrane protein extracted, which were subsequently normalized. Samples were resolved on a 12% SDS–PAGE gel and transferred to a polyvinylidene fluoride membrane. The membranes were blocked overnight in PBST buffer [10 mM phosphate-buffered saline (PBS, pH 7.4), 0.05% Tween-20] supplemented with 5% nonfat milk at 4°C. The membranes were then incubated with OmpT mouse-origin monoclonal antibody for detection and then with secondary antibodies [goat anti-mouse IgG conjugated with horseradish peroxidase (Beyotime, China)]. Finally, the membranes were exposed to a chemiluminescent HRP substrate (Sharebio, China).

### 2.6 Protein expression and purification of RNase 7

RNase 7 was expressed in *E. coli* BL21(AI) cells harboring the expression plasmid pDEST17-RNase 7. Cultures were induced with L-arabinose (final concentration 2%) in LB broth medium for 3 h. The cell pellet was collected by centrifugation and resuspended in 20 mL buffer D (20 mM Tris-Cl, pH 7.5). After cell lysis by sonication, the supernatant was collected by centrifugation and purified through a Ni-NTA column (eluent: 50 mM Na_2_HPO_4_, 0.3 M NaCl, and 250 mM imidazole, pH 8.0) and a Superdex-200 column (buffer E: 20 mM Tris–HCl, 150 mM NaCl, and 10% glycerol, pH 8.0). RNase 7 was stored in buffer E (20 mM Tris–HCl, 150 mM NaCl, 10% glycerol) at 4°C. The protein concentration, determined by the BCA method, was approximately 0.15 mg/mL.

### 2.7 Growth kinetics of bacteria co-incubated with protamine

To study the growth kinetics of bacteria incubated with protamine, we cultivated bacteria in N-minimal media to avoid potential interference from some components of complex media on the function of OmpT. Overnight bacterial cultures in N-minimal medium were prepared and centrifuged. The cultures were resuspended in 10 mL of fresh N-minimal medium and normalized to an OD_600_ of 0.35 (a final concentration of 3.5 × 10^8^ colony forming units [CFU]/mL), and protamine was then added to the bacterial suspension at a final concentration of 100 μg/mL. The mixture was shaken at 37°C, and the OD_600_ was measured every 2 h. The results were drawn into the growth curve of bacteria, and GraphPad Prim 7 software was used for differential analysis.

### 2.8 Proteolytic cleavage of AMPs

Bacterial cells grown in N-minimal medium to an OD_600_ nm of 0.6–0.8 were washed, pelleted by centrifugation, resuspended in PBS (pH 7.4), and normalized to a bacterial density of 3 × 10^10^ CFU/mL. Bacteria were combined at a 1:4 (v/v) ratio with 2.5 μg/μL protamine or at a 1:12 (v/v) ratio with 0.15 μg/μL RNase 7 to facilitate visualization of degradation products and incubated at 37°C for various time points. Bacteria were separated from peptide cleavage products by centrifugation, and supernatants were mixed with 2× Tricine sample buffer (Beyotime, China) or 5× SDS-PAGE protein loading buffer (Yeasen Biotechnology, China), then boiled and stored at −20°C. Peptide cleavage products were heated at 96°C for 10 min and separated by 16.5% Tris-Tricine SDS-PAGE (Beyotime, China) or 13% SDS-PAGE. After fixation for 30 min in 5% glutaraldehyde and subsequent washing for 30 min with deionized water, the peptides were stained for 1 h with Coomassie blue G-250.

### 2.9 Fluorescence resonance energy transfer (FRET) activity assay

The synthetic FRET substrate containing ortho-aminobenzoic acid (Abz) as the fluorophore and group 2, 4-nitrophenyl (Dnp) as the quencher and a dibasic motif (RK) in its center (2Abz-SLGRKIQI-K(Dnp)-NH2) was purchased from GL Biochem Ltd. (China)^1^ (25, 49). To perform the assay, bacteria were grown in N-minimal medium to the mid-exponential phase and normalized to an OD_600_ of 0.6–0.8. The bacterial cells were centrifuged, resuspended in PBS (pH 7.4), and normalized to 3 × 10^8^ CFU/mL. Bacteria (~2.25× 10^7^ CFU in 75 μL) were mixed in a 96-well plate with 75 μL of FRET substrate (final concentration 40 μM). The fluorescence emission was monitored for 360 min at 25°C using a BioTek Synergy 2 plate reader with an excitation wavelength of 325 nm and an emission wavelength of 430 nm. The initial background measurements were subtracted from the final reaction sample values. The kinetic parameters (*K*_m_, *K*_cat_, and *K*_cat_/*K*_m_) were calculated by measuring OmpT activity at 0–240 μM substrate and fitting the resulting Michaelis–Menten equation.

### 2.10 Colorimetric assay

Samples of membrane fractions were diluted in buffer A to appropriate concentrations prior to measurements of OmpT activity. OmpT activity was assessed in a coupled spectrophotometric assay using the chromogenic substrate IAA-Arg-Arg-pNA purchased from GL Biochem Ltd. (China) (49). In a 200 μL reaction system, the assay included 100 μg total outer membrane protein, 0.5 mM IAA-Arg-Arg-pNA, 1 mM Tween 20, 20 mM Mes (pH 7.0), and 0.5 U·mL^−1^ aminopeptidase M. OmpT specific cleavage between the two arginines results in the release of Arg-pNA, which is subsequently cleaved by aminopeptidase M (Sigma, USA), present in excess to ensure its activity is not rate-limiting. This process releases pNA, detected spectrophotometrically at 405 nm over 12 h at 37°C using a BioTek Synergy 2 plate reader. Initial background measurements were subtracted from the final reaction sample values. The data from these experiments were plotted on a kinetic curve and analyzed using GraphPad Prism 7 software.

### 2.11 Inhibition of proteolytic activity

To investigate the inhibition of proteolytic activity, three serine protease inhibitors [PMSF (Beyotime, China), leupeptin (Beyotime, China), and aprotinin (all from Beyotime, China)] were used to study the roles of Asp^267^ (aspartic acid, D) and Ser^276^ (serine, S) in cOmpT through a FRET activity assay. Bacteria grown in N-minimal medium to mid-exponential phase were normalized to an OD_600_ nm of 0.6–0.8. Bacterial cells were pelleted by centrifugation, resuspended in PBS (pH 7.4), and adjusted to a bacterial density of 3 × 10^8^ CFU/mL. In a 175 μL reaction system within a 96-well plate, the assay included bacteria (~2.25 × 10^7^ CFU), 40 μM FRET substrate, and either 0.8 mM PMSF, 0.8 mM leupeptin, or 1 mM aprotinin. Fluorescence (with an excitation of 325 nm and an emission of 430 nm) was monitored over 360 min at 25°C using a BioTek Synergy 2 plate reader. Initial background measurements were subtracted from the final reaction sample values. The data were used to construct kinetic curves, and GraphPad Prism 7 software was used for differential expression analysis.

### 2.12 Molecular docking

The molecular docking method was used to simulate the interactions between protamine and cOmpT/pOmpT and their mutants by using the software Autodock (50). The crystal structure of cOmpT (PDB ID: 1I78) was downloaded from the PDB.^2^ The structures of the pOmpT, cOmpT_D267S/S276T_, and pOmpT_S267D/T276S_ mutants were all obtained by online modeling using Swiss-Model based on the crystal structure of cOmpT.^3^ Protamine (P69015) is based on the predicted structure from the Alpha Fold protein structure database.^4^ Autodock Tools software was used to delete water, add hydrogens, compute Gasteiger charges, and assign AD4-type atoms to them. The grid box was 126 × 126 × 126 with a grid point spacing of 0.7 Å and centered at 25.946, 58.190, and 13.075 (x, y, z). AutoDock was used to dock protamine with cOmpT, pOmpT, and their double mutants, and the potential binding conformations were identified using a genetic algorithm. The genetic algorithm parameters included a population size of 150, a maximum number of media of 2,500,000, a maximum number of generations of 27,000, a GA crossover mode of twopt, and a number of GA runs of 10. A scoring function was used to evaluate the calculated binding free energy, and clustering was carried out using the root mean square error (RMSD) between the binding modes. The least energetic conformation was chosen as the optimal binding mode for OmpT and protamine.

### 2.13 Animal experiment

This study received approval from the Institutional Animal Care and Use Committee (IACUC) of Yangzhou University (Chicken: SCXK(Su)2021–0027) and was conducted in accordance with the Animal Ethics Procedures and Guidelines of the People’s Republic of China.

The LD_50_ assay was conducted on 3-day-old specific pathogen-free (SPF) chickens (White Leghorn; Jinan SPAFAS Poultry Co., Ltd., Jinan, China) to assess the pathogenicity of the wild-type strain E058 and its mutant strains in the c*ompT* and p*ompT* genes. Cultures of the wild-type strain and its mutant derivatives were grown to the logarithmic phase at 37°C. The bacteria from each strain were collected, washed twice, and suspended in sterile PBS (containing 10% glycerol) before being diluted to appropriate concentrations (10^10^ or 10^9^ CFU/mL) and then further diluted to 10^9^, 10^8^, 10^7^, 10^6^, 10^5^, 10^4^, and 10^3^ CFU/mL, respectively. Six birds in each group were challenged via the air sac with 0.1 mL of each culture suspension. The mock group was injected with sterile PBS. The chickens were monitored for 7 days until survival rates stabilized. LD_50_ results were estimated using the Reed-Muench method and IBM SPSS statistics software. Statistical significance was assessed with the t-test, and differences with *p*-values <0.05 were considered statistically significant.

## 3 Results

### 3.1 pOmpT is a homologous derivative of cOmpT

**Figure 1:**
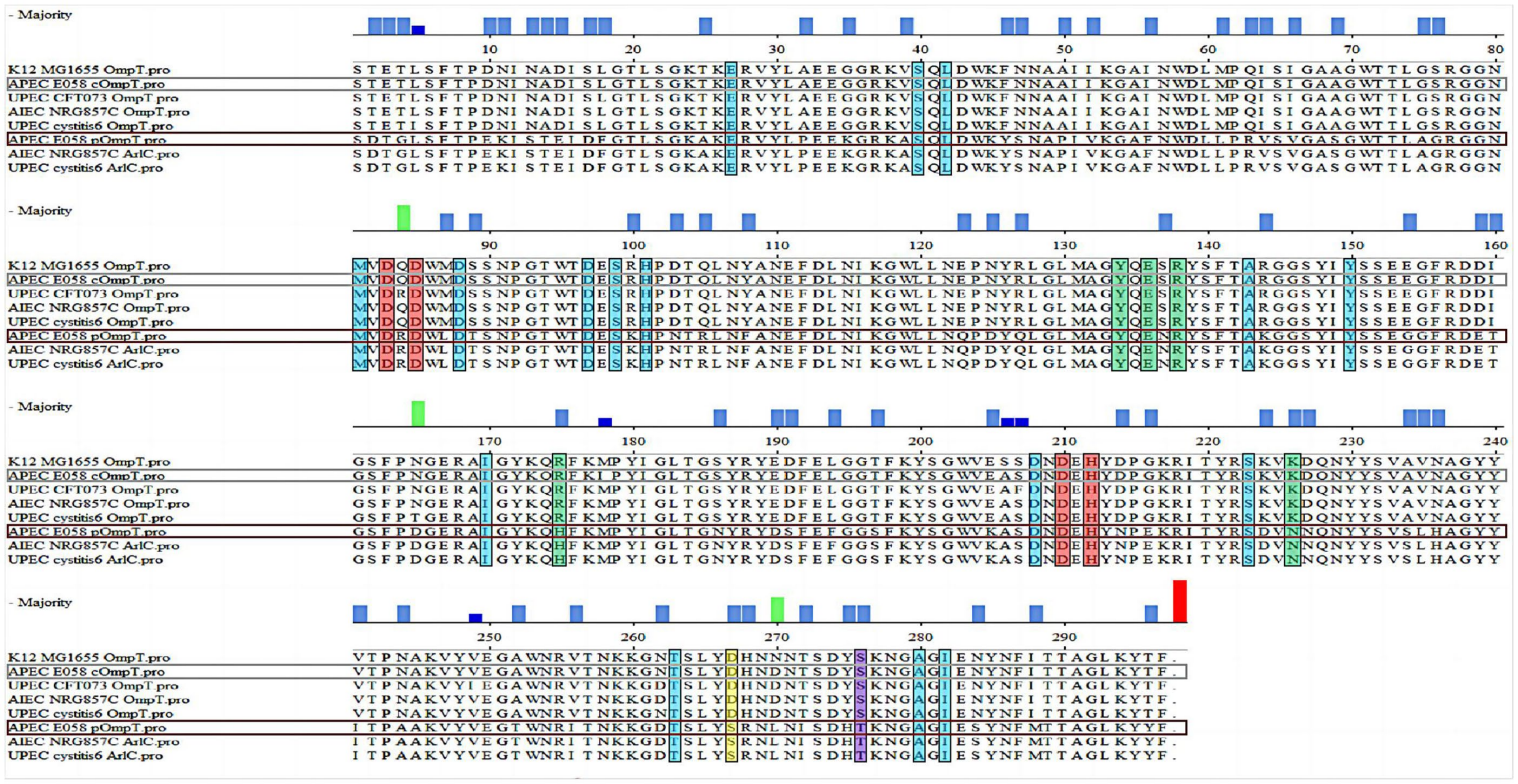
pOmpT is a homologous derivative of cOmpT. Alignment of the amino acid sequences between OmpT-like protein from different strains. The sequences of both the chromosome-encoded OmpT (E058 cOmpT) and the plasmid-encoded OmpT (E058 pOmpT) in the APEC E058 strain were determined by our laboratory; In the figure, the residues marked in the red boxes are the catalytic residues of OmpT outer membrane protease, the residues marked in the blue boxes are the enzyme active sites of OmpT and the green boxes indicated the amino acid residues as the binding site of OmpT and LPS.

Sequence alignment revealed that APEC E058 pOmpT shared a highly homologous amino acid sequence with those of cOmpT from UPEC CFT073 and cystitis 6, adherent-invasive *E. coli* (AIEC) NRG857C, and APEC E058 (≈ 76%) (Figure 1). Notably, pOmpT of E058 exhibited 226 out of 297 amino acid sequence identities with those of cOmpT, while it shared an identical sequence identity (297/297) with those of the ArlC encoded by plasmids in both UPEC cystitis 6 and AIEC NRG857C (Figure 1), indicating a common ancestral origin for these plasmid-encoded OmpTs. The alignment suggested that E058 cOmpT is a typical outer membrane protease, similar to OmpT of *E. coli* K12 MG1655, UPEC CFT073, AIEC NRG857C, and UPEC cystitis 6 strains. Although pOmpT shared only about 76% sequence identity with cOmpT, the critical residues of OmpT protease between E058 pOmpT and E058 cOmpT, including the catalytic and active sites, are identical. Nonetheless, two residues in the LPS-binding sites of E058 pOmpT differed from those in cOmpT strains. In addition, AlphaFold 3.0 was used to predict the structure of pOmpT based on the amino acid sequence of E058 pOmpT, indicating both cOmpT and E058 pOmpT exhibited a β-barrel structure.

**Figure 2:**
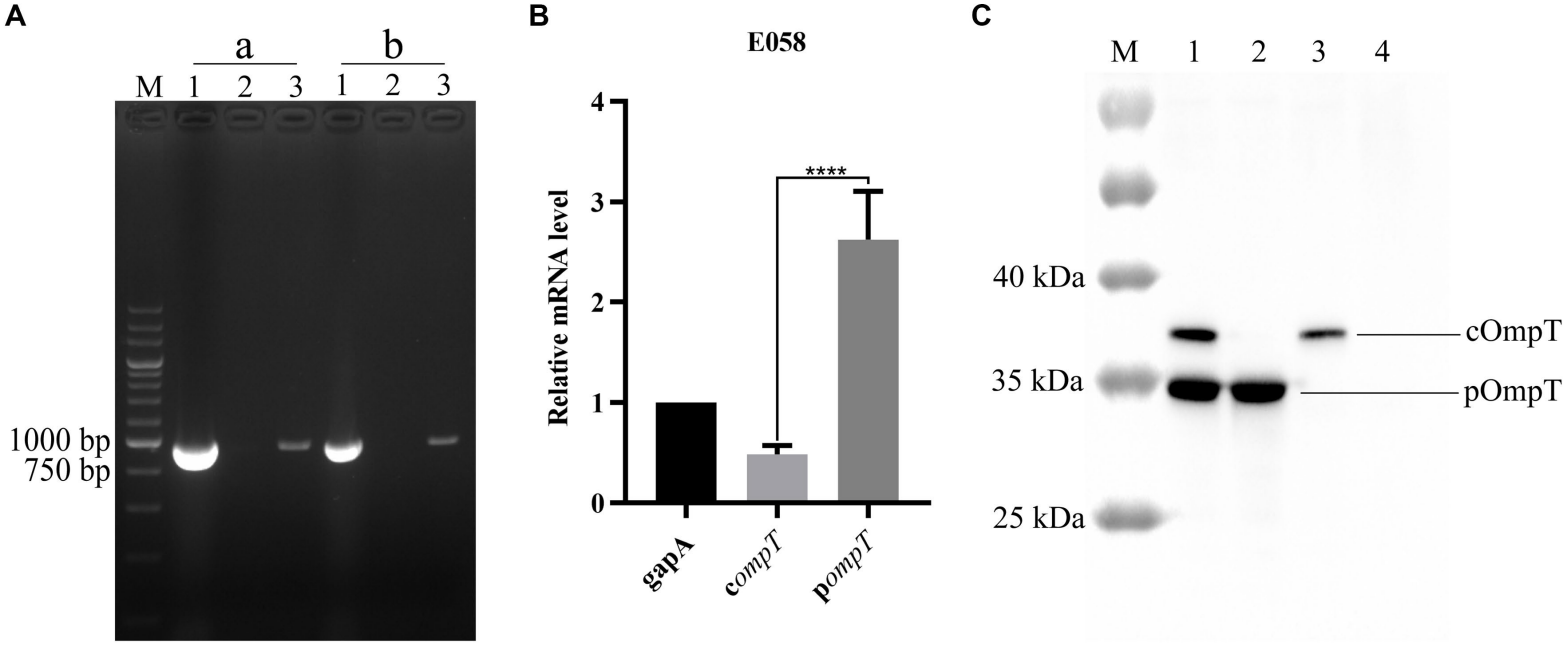
pOmpT is an outer membrane protein. **(A)** RT-PCR analysis of c*ompT* and p*ompT* genes in APEC E058 strain. a: c*ompT* gene; b: p*ompT* gene; Lane 1: Genomic DNA from E058; Lane 2: Total RNA from E058; Lane 3: cDNA derived from the total RNA of E058. 200 bp DNA marker (TaKaRa) was used as the molecular size standard (lane M). **(B)** qPCR analysis of the transcription level of c*ompT* and p*ompT* genes in APEC E058 strain. **(C)** The extracted total outer membrane proteins were also analyzed with OmpT specific monoclonal antibody. Lane 1: Total outer membrane protein extracted from strain E058; Lane 2: Total outer membrane protein extracted from strain E058Δc*ompT*; Lane 3: Total outer membrane protein extracted from strain E058Δp*ompT*; Lane 4: Total outer membrane protein extracted from strain E058Δc*ompT*Δp*ompT*; PageRuler™ prestained protein ladder (Thermo Fisher scientific, USA) was used as the molecular size standard (lane M). All experiments were repeated three times. Statistical significance was determined using the *t*-test. Differences with *p*-values <0.05 were considered as statistically significant. ****p < 0.0001.

Reverse transcription PCR (RT–PCR) revealed that both the c*ompT* and p*ompT* genes can be transcribed constitutively and are 954 bp in length in the APEC E058 strain (Figure 2A). The results of quantitative PCR showed that the transcription level of the p*ompT* gene was significantly greater than that of the c*ompT* gene (Figure 2B). It is unclear whether pOmpT is the same as cOmpT and is located in the outer membrane, although it shares the same number of nucleotides and highly similar amino acid sequence as cOmpTs up to about 76 percent and is also transcribed in E058. To determine the location of pOmpT, the outer membrane proteins of the wild-type strain E058 and the c*ompT*/p*ompT* single/double-gene deletion mutants were extracted and detected by western blotting using an OmpT specific monoclonal antibody. The results showed that a single band could be detected in the E058 c*ompT*/p*ompT* single-gene deletion mutants, and the sizes of the bands differed between the two strains, suggesting that two different proteins were expressed in these two deletion mutants (Figure 2C). Moreover, two bands in the wild-type strain E058 were detected, and the positions of the bands were consistent with those observed in the E058 c*ompT*/p*ompT* single-gene deletion mutants (Figure 2C). The above results indicated that both pOmpT and cOmpT are expressed on the outer membrane of E058. In addition, the estimated protein molecular weight of pOmpT (≈36 kDa) was less than that of cOmpT (≈37 kDa) based on their amino acid sequences. Moreover, the expression level of pOmpT on the membrane was found to be higher than that of cOmpT, consistent with qPCR results.

### 3.2 pOmpT cannot resist protamine

**Figure 3:**
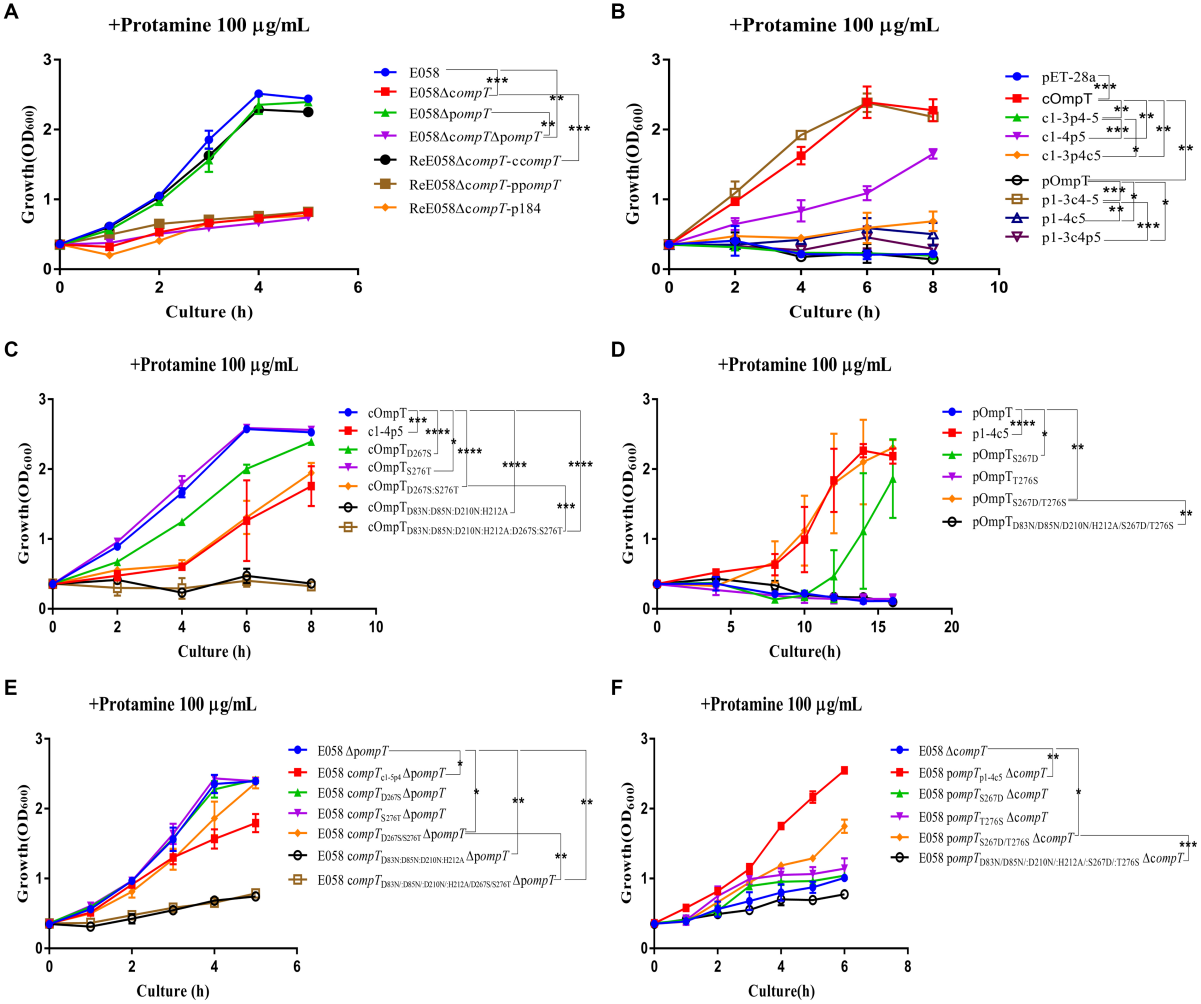
The kinetics of bacterial growth under protamine treatment. **(A)** Growth profiles of APEC wild-type strain E058, c*ompT*/p*ompT* single/double-gene deletion strains and their complementation strains under protamine treatment. **(B)** Growth profiles of strains *E. coli* BL21(DE3) expressing cOmpT, pOmpT and the chimeric protein with the interchanged different loops between cOmpT and pOmpT under protamine treatment. **(C)** Growth profiles of strains *E. coli* BL21(DE3) expressing site-directed mutagenesis of cOmpT under protamine treatment. **(D)** Growth profiles of strains *E. coli* BL21(DE3) expressing site-directed mutagenesis of pOmpT under protamine treatment. **(E)** Growth profiles of p*ompT* gene deletion strains expressing site-directed mutagenesis of cOmpT of APEC E058 under protamine treatment. **(F)** Growth profiles of c*ompT* gene deletion strains expressing site-directed mutagenesis of pOmpT of APEC E058 under protamine treatment. The bacterial strains used here are listed in Supplementary Table S1. All experiments were repeated three times. Statistical significance was determined using the two-way ANOVA. Differences with *p*-values <0.05 were considered as statistically significant. *p < 0.05; **p < 0.01; ***p < 0.001; ****p < 0.0001.

Protamine cleavage is one of the important roles for cOmpT in host resistance (13, 34–36). To understand the role of pOmpT in resisting the host, growth kinetics were evaluated for the wild-type strain E058, c*ompT*/p*ompT* single/double-gene deletion strains and complementation strains incubated with protamine. Compared with that of the wild-type strain, the growth of the strains E058Δp*ompT* and ReE058Δc*ompT-*cc*ompT* did not significantly differ when cOmpT was present in the strains (*p* > 0.05), while the growth ability of the strain was significantly reduced when only pOmpT was present in the deletion strain E058Δc*ompT* and complementation strain ReE058Δc*ompT-*pp*ompT* (*p* < 0.01) (Figure 3A). The same phenomenon was verified in recombinant c*ompT*/p*ompT* gene-expressing bacteria (Figure 3B), which indicates that cOmpT can resist protamine but that pOmpT cannot.

### 3.3 The difference in the kind of residues at positions 267 and 276 discriminates protamine cleavage specificity by cOmpT and pOmpT

To explore which one of the five loops in pOmpT plays a critical role in protamine deactivation, mutants of pOmpT and cOmpT were engineered by swapping the loops between the two. The sensitivity of these modified constructs to protamine was assessed. The findings revealed a significant decrease in protamine resistance for the cOmpT variant containing the pOmpT L5 segment (Figure 3B). Conversely, replacing the L5 region of cOmpT with that of pOmpT in the pOmpT construct substantially increased its protamine resistance (Figure 3B). Further experimentation with mutants involving other loop exchanges between cOmpT and pOmpT indicated no significant impact on protamine resistance, underscoring the pivotal role of the L5 region in mediating the differences in protamine resistance between cOmpT and pOmpT. In pursuit of identifying crucial residues in cOmpT’s L5 contributing to protamine resistance, exchanges of single or multiple L5 residues between cOmpT and pOmpT were conducted. Protamine resistance assays demonstrated that replacing Ser^276^ in cOmpT with Thr^276^ from pOmpT significantly reduced protamine resistance (*p* < 0.05) (Figure 3C). Substituting Asp^267^ in cOmpT with Ser^267^ from pOmpT, or concurrently swapping both Asp^267^ and Ser^276^ in cOmpT with Ser^267^ and Thr^276^ from pOmpT, resulted in a marked decrease in protamine resistance (*p* < 0.001) (Figure 3C). Compared to pOmpT, a mutant form pOmpT_S267D_ exhibited notably enhanced growth in the presence of protamine resistance (*p* < 0.05), and the mutant pOmpT_S267D/T276S_ showed significantly increased protamine resistance (*p* < 0.01) (Figure 3D). The growth kinetics of bacterial resistance to protamine further affirmed the contribution of residues 267 and 276 in cOmpT toward protamine resistance in APEC E058 (Figures 3E,F).

We also analyzed protamine cleavage efficiency in *E. coli* expressing cOmpT, pOmpT and their various mutants. The cleavage efficiency of protamine by single-mutant D267S and double-mutant D267S/S276T of cOmpT was significantly lower than that of cOmpT (*p* < 0.001) (Supplementary Figures S1A,C). In contrast, the cleavage efficiency of protamine by the single mutant T276S and double mutant S267D/T276S of pOmpT increased significantly (*p* < 0.01) (Supplementary Figures S1B,D). Notably, the cleavage efficiency of the single mutant S267D of pOmpT was even greater than that of the other mutants (*p* < 0.001) (Supplementary Figures S1B,D). The above results indicated that L5 of cOmpT is the key loop that contributes to differences in resistance to protamine between cOmpT and pOmpT, and the key residues are residues 267 and 276.

To investigate the potential interaction between OmpT and protamine, protamine was docked to cOmpT, pOmpT, and their mutants using AutoDock software (AutoDock 4.0, Scripps Research Institute, USA)^5^ (50). The configuration with the lowest binding energy underwent visual analysis (51). Given that cOmpT and pOmpT are outer membrane proteins, simulation models representing the transmembrane region were excluded (50). Docking simulation results indicated that protamine could not dock into the binding pocket of pOmpT, while it readily docked into that of cOmpT (Supplementary Figures S2A,B). In the cOmpT model, six residues—D159, I160, K216, D267, S273, and D274—were found to form hydrogen bonds with corresponding residues of protamine, highlighting their importance for protamine’s binding to cOmpT (Supplementary Figure S2B). However, the cOmpT_D267S/S276T_-protamine complex revealed only three anchor residues (E34, D270, and N269) for protamine binding (Supplementary Figures S2C,D), aligning with the diminished cleavage ability of the cOmpT_D267S/S276T_ mutant toward protamine. Furthermore, replacing residues 267 and 276 in pOmpT with those from cOmpT enabled protamine docking into its binding site, forming hydrogen bonds at three residues, including D267 (Supplementary Figures S2E,F). This suggests that D267 in cOmpT is a key residue for anchoring protamine.

### 3.4 Residues 267 and 276 of cOmpT and pOmpT are involved in substrate affinity

**Figure 4:**
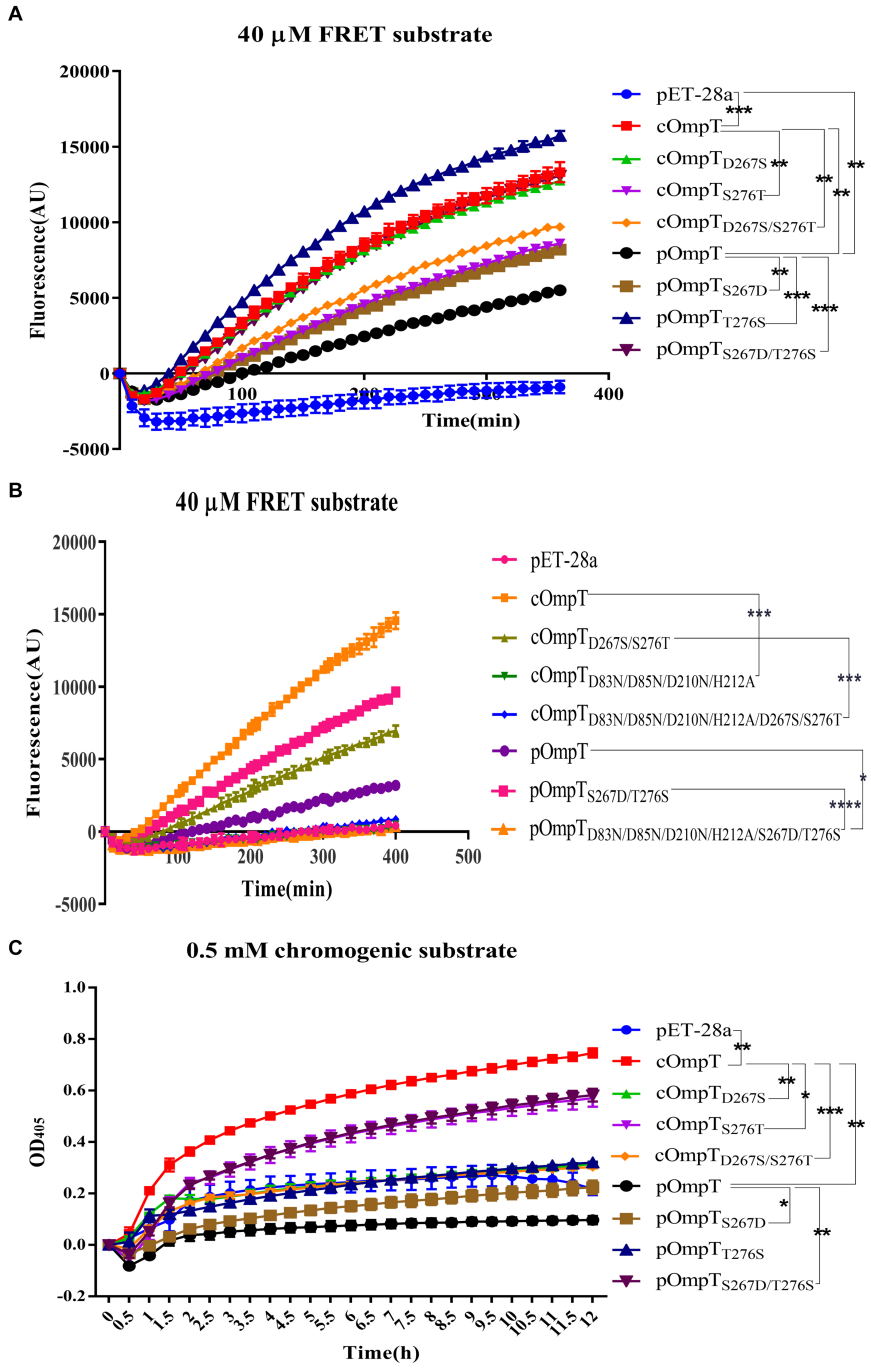
Determination of enzyme activity of cOmpT, pOmpT and its site-directed mutants. **(A,B)** Enzyme activity of cOmpT, pOmpT and their variants expressed in *E. coli* BL21(DE3) by utilizing a FRET substrate (2Abz-SLGRKIQI-K(Dnp)-NH2). **(C)** Enzyme activity of cOmpT, pOmpT and their variants expressed in *E. coli* BL21(DE3) by utilizing a chromogenic substrate (IAA-Arg-Arg-pNA). Statistical significance was determined using the two-way ANOVA. Differences with *p*-values <0.05 were considered as statistically significant. *p < 0.05; **p < 0.01; ***p < 0.001; ****p < 0.0001.

OmpT specifically cleaves substrates at dibasic motifs (RR, RK, and KK) using synthetic peptides as substrates (31, 32). Protamine, due to its abundance of dibasic motifs in the primary structure, is cleaved by cOmpT, explaining why the protamine cleavage products in gels are not a single band. Synthetic peptides containing dibasic motifs, such as the synthetic FRET substrate [2Abz-SLGRKIQI-K(Dnp)-NH2] and chromogenic substrate [IAA-Arg-Arg-pNA], are commonly used to detect OmpT enzyme activity (25, 49). We examined whether cOmpT and pOmpT share similar characteristics and if differences at residues 267 and 276 affect their activity toward synthetic substrate. Digestion results showed that both pOmpT and cOmpT in equal amounts of *E. coli* BL21(DE3) could cleave the synthetic substrate (Figures 4A–C). Although the cleavage efficiency of cOmpT remains higher than that of pOmpT, this differs from the cleavage activity toward the substrate protamine, which is exclusively digested by cOmpT. Single and double replacement mutations of cOmpT (S276T and D267S/S276T) significantly decreased cOmpT activity in equal amounts of *E. coli* BL21(DE3) (*p* < 0.01). In contrast, replacement mutation of pOmpT significantly increased cleavage efficiency compared to pOmpT (*p* < 0.001) in equal amounts of *E. coli* BL21(DE3) (Figure 4A). The notable efficiency in cleaving substrates by the pOmpT_S276T_ variant underscores the critical role of residue 276. This observation further supports the contribution of residues 267 and 276 to the cleavage differences observed between cOmpT and pOmpT.

**Figure 5:**
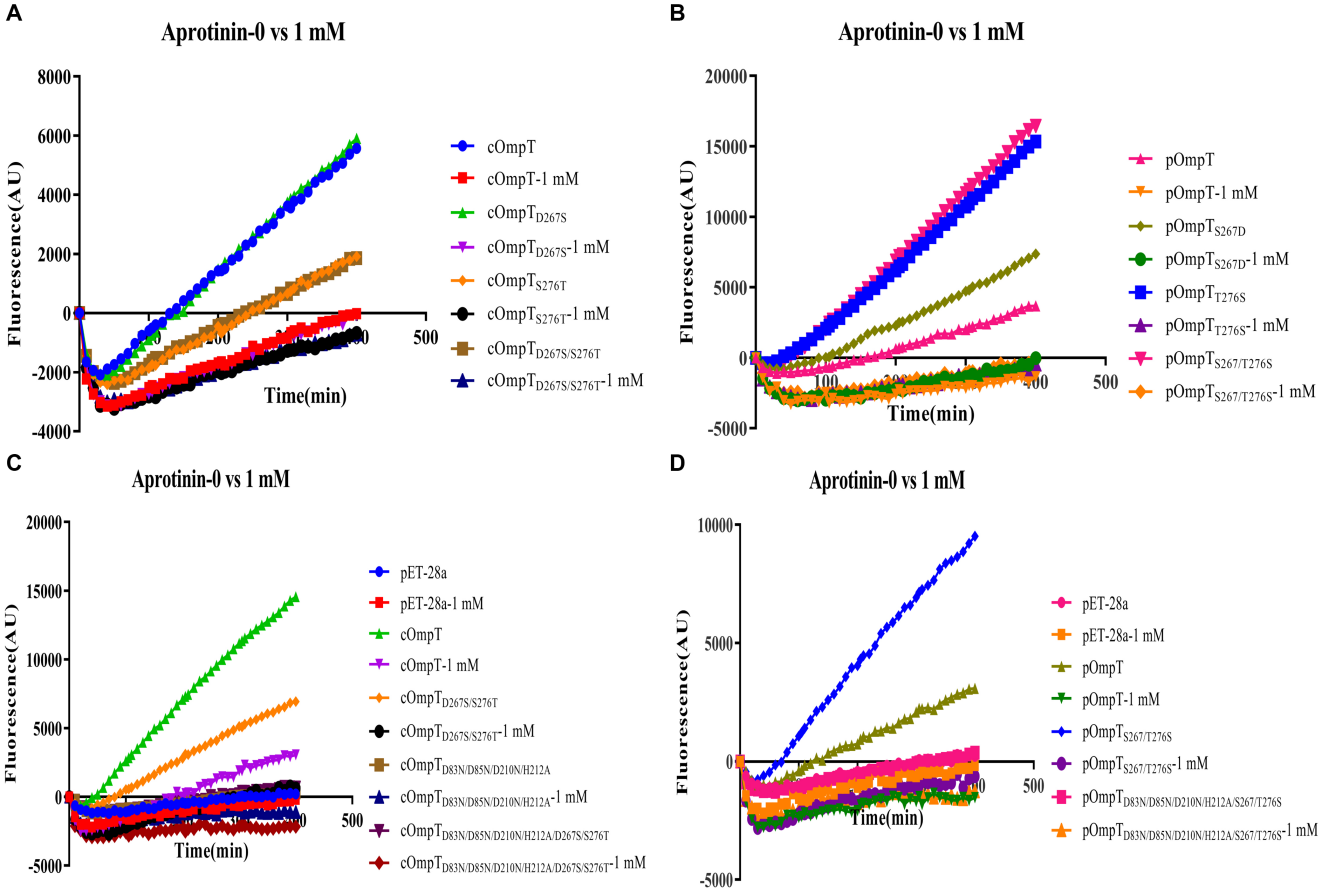
Inhibition of enzyme activity of cOmpT, pOmpT and its site-directed mutants expressed in *E. coli* BL21 by aprotinin. **(A–D)** FRET assays were performed with cOmpT, pOmpT and their site-directed mutants in PBS or in the presence of aprotinin (1 mM).

It has been documented that cOmpT can be inhibited by serine protease inhibitors (27, 52). Experiments were conducted to ascertain whether pOmpT is similarly affected by serine protease inhibitors as cOmpT. The findings demonstrated that pOmpT in *E. coli* BL21(DE3) was inhibited by aprotinin (Figure 5) and leupeptin (Supplementary Figure S3), along with mutants of residues 267 and 276 in both cOmpT and pOmpT variants (Figure 5; Supplementary Figure S3). This indicates that the difference between pOmpT and cOmpT does not influence their inhibition by serine protease inhibitors. Residues Asp^83^, Asp^85^, Asp^210^, and His^212^ have been proposed as the catalytic sites for cOmpT (24, 25). Mutations at these sites, along with mutations at residues 267 and 276 in both cOmpT and pOmpT, resulted in a loss of activity toward synthetic substrates, both with and without the presence of inhibitors. This suggests these residues are essential for the activity of pOmpT as well (Figures 4B; 5C,D; Supplementary Figures S3C,D; S4C,D). Furthermore, it implies that residues 267 and 276 may not act as catalytic sites in either protein variant. To elucidate the roles of residues 267 and 276 further, the *K*_m_ values of cOmpT, pOmpT, and their respective mutants were determined. The *K*_m_ values for the mutants of cOmpT were found to be higher than those of the wild type (1.56–4.44 times) (Table 1), indicating a decrease in substrate binding affinity. Conversely, the *K*_m_ value of pOmpT was higher than that of its mutants (2.37–4.24 times) (Table 1), suggesting that residues 267 and 276 are involved in substrate binding rather than in catalysis. The *K*_cat_/*K*_m_ values for cOmpT and pOmpT and their mutants further revealed that the catalytic efficiency of cOmpT is significantly higher than that of pOmpT. Mutations at residues 267 and 276 improved the catalytic efficiency of pOmpT, whereas they dramatically decreased that of cOmpT (Table 1), indicating that changes in binding affinity due to these residues also affect catalytic efficiency.

**Table 1:** Kinetic parameters of (c/p)OmpT and their site-directed mutants expressed in *E. coli* BL21 using synthetic fluorimetric peptide as substrate.

Strains	*k*_cat_ (min^−1^)	*K*_m_ (nM)	*k*_cat_/*K*_m_ (nM·min^−1^)
cOmpT	394.87	10.70	3.69 × 10^7^
cOmpT_D267S_	341.00	16.70	2.04 × 10^7^
cOmpT_S276T_	437.60	47.50	9.21 × 10^6^
cOmpT_D267S/S276T_	517.73	40.80	1.27 × 10^7^
pOmpT	430.40	66.70	6.46 × 10^6^
pOmpT_S267D_	285.40	28.10	1.02 × 10^7^
pOmpT_T276S_	405.27	15.70	2.58 × 10^7^
pOmpT_S267D/T276S_	478.47	21.50	2.23 × 10^7^

### 3.5 Residues 267 and 276 of pOmpT contribute to the cleavage of human RNase 7

**Figure 6:**
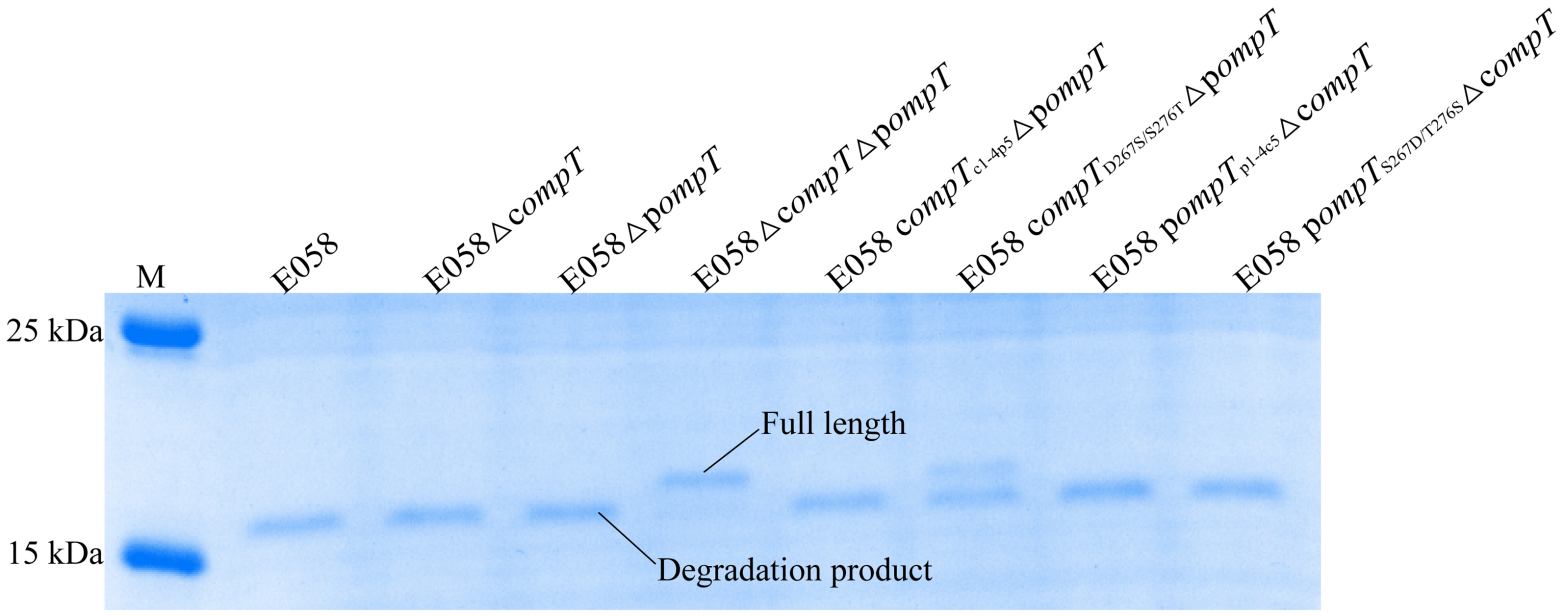
Changes of residues at positions 267 and 276 of pOmpT can also change its ability to cleave human RNase 7. RNase 7 was incubated with APEC E058 expressing cOmpT, pOmpT or their mutants for 15 min. 180 kDa Prestained Protein Marker (Vazyme, China) was used as the protein molecular size standard (lane M).

ArlC in UPEC, which shares 100% identity with pOmpT, can promote bacterial resistance to the host by cleaving the large molecule AMP human RNase 7 (23). As expected, RNase 7 was cleaved with a clear cleavage product band remaining (<17.7 kDa) when incubated with the strain expressing pOmpT (such as strains E058 and E058Δc*ompT*). However, Strain E058Δc*ompT*Δp*ompT* without c/pOmpT did not cleave RNase 7 with a clear RNase 7 protein band remaining (~17.7 kDa), indicating that pOmpT can also cleave RNase7 (Figure 6). Interestingly, RNase 7 was also cleaved by cOmpT (strain E058Δp*ompT*), which was shown to have no effect on RNase 7 in UPEC isolate cystitis 6 (23). Synthetic substrate digestion showed that residues 267 and 276 of c/pOmpT are involved in substrate recognition (Figure 6; Table 1), so we also checked the influence of changes in residues 267 and 276 on the proteolytic activity of c/pOmpT on RNase 7. The results showed that the replacement of residues 267 and 276 of cOmpT with that of pOmpT dramatically decreased the activity of cOmpT on RNase 7, and a clear band of intact RNase 7 was observed compared with that of cOmpT (Figure 6).

### 3.6 Residues 267 and 276 of cOmpT and pOmpT are closely related to APEC pathogenicity

**Figure 7:**
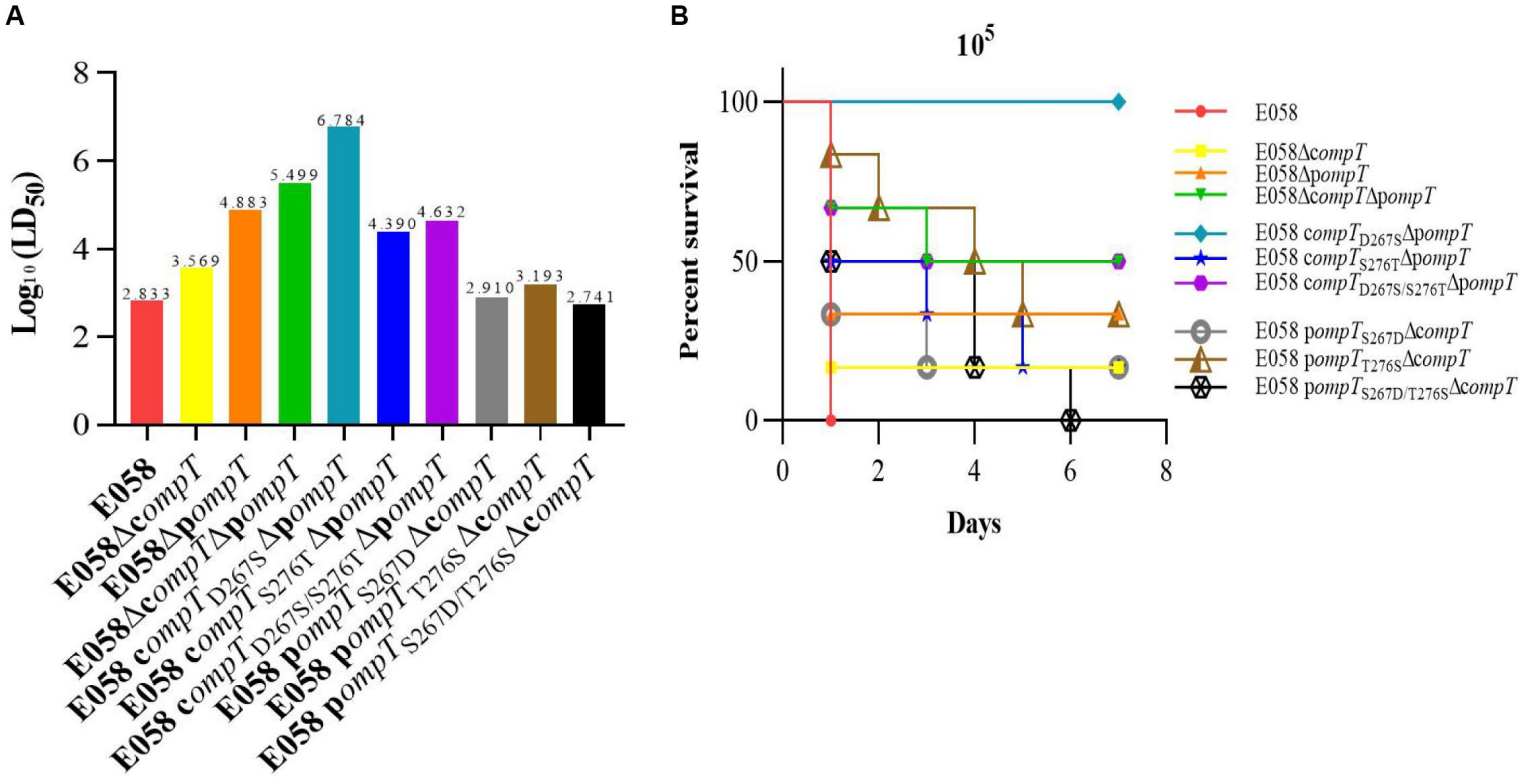
Residues 267 and 276 of cOmpT and pOmpT are closely related to APEC pathogenicity. **(A)** LD_50_ in a chicken infection model was used to assess the effect of cOmpT, pOmpT, and their site-directed mutants on the pathogenicity of APEC E058. The data of LD_50_ for all strains were presented in Table 2. **(B)** The survival of birds challenged with each strain at a dose of 10^5^ CFU/bird was monitored for 7 days post-challenge based on the Kaplan Meyer survival curves.

The 50% lethal dose (LD_50_) in a chicken infection model was used to assess the pathogenicity of cOmpT, pOmpT, and their site-directed mutants in APEC E058. The LD_50_ for all strains is presented in Table 2 and Figure 7. The pathogenicity of single or double-gene deletion strains of cOmpT and pOmpT in APEC E058 was significantly reduced compared to the wild-type strain E058, by almost 5 (10^3.569^/10^2.833^, *p* < 0.05) (cOmpT single deletion), 112 (10^4.883^/10^2.833^, *p* < 0.01) (pOmpT single deletion), and 463 (10^5.499^/10^2.833^, *p* < 0.001) (cOmpT and pOmpT double deletion) times, respectively. This confirms the role of cOmpT and pOmpT as virulence factors in the pathogenicity of APEC E058. Interestingly, the LD_50_ of the mutant strain E058 c*ompT*_D267S_Δp*ompT* was significantly lower, almost 80 times less than the single-gene deletion strain E058Δp*ompT* (10^6.784^/10^4.883^, *p* < 0.01), indicating that residue 267 of cOmpT contributes more to APEC pathogenicity than residue 276. Conversely, replacing residues 267 and 276 of pOmpT with those of cOmpT in c*ompT* gene deletion strains resulted in an LD_50_ higher than that of the single-gene deletion strain E058Δc*ompT*. The double substitution of residues 267 and 276 (10^2.741^) exhibited the highest virulence, almost equivalent to the wild-type strain E058 (10^2.883^/10^2.741^, *p* > 0.05). Following this, the substitution of residue 267 (10^2.910^) and residue 276 (10^3.193^) occurred, respectively. This observation underscores a significant phenomenon: upon the deletion of cOmpT in E058, replacing only 2 residues of pOmpT resulted in its virulence matching that of the parental strain. Put differently, the substitution of two residues not only preserved the pathogenicity of pOmpT itself but also fully compensated for the virulence of cOmpT. These findings suggest that residues 267 and 276 of pOmpT also play an essential role in APEC virulence.

**Table 2:** LD_50_ of wild type strain and their mutant strains.

Strains	Deaths/birds Inoculated								LD_50_
	Inoculated Doses (CFU/bird)								
	10^9^	10^8^	10^7^	10^6^	10^5^	10^4^	10^3^	10^2^	
E058	-	-	-	6/6	6/6	4/6	4/6	2/6	10^2.833^
E058Δc*ompT*	-	-	-	6/6	5/6	3/6	4/6	0/6	10^3.569^
E058Δp*ompT*	-	-	6/6	6/6	4/6	1/6	0/6	-	10^4.883^
E058Δc*ompT*Δp*ompT*	-	6/6	6/6	3/6	3/6	0/6	-	-	10^5.499^
E058 c*ompT*_D267S_Δp*ompT*	6/6	6/6	3/6	1/6	0/6	-	-	-	10^6.784^
E058 c*ompT*_S276T_Δp*ompT*	-	-	6/6	6/6	5/6	2/6	1/6	-	10^4.390^
E058 c*ompT*_D267S/S276T_Δp*ompT*	-	-	6/6	6/6	3/6	2/6	0/6	-	10^4.632^
E058 p*ompT*_S267D_Δc*ompT*	-	-	-	6/6	5/6	4/6	2/6	4/6	10^2.910^
E058 p*ompT*_T276S_Δc*ompT*	-	-	-	6/6	4/6	4/6	4/6	2/6	10^3.193^
E058 p*ompT*_S267D/T276S_Δc*ompT*	-	-	-	6/6	6/6	6/6	2/6	2/6	10^2.741^

“-” was defined as “not done”.

## 4 Discussion

Omptins are key virulence factors involved in several gram-negative bacterial pathogens (14, 15, 17, 39). Numerous studies have suggested that OmpT-like proteins are present in intestinal or extraintestinal pathogenic *E. coli* strains that infect humans and animals and that they enhance bacterial virulence by participating in the cleavage of host AMPs (12, 13, 23, 41). Their contribution to *E. coli* virulence mainly depends on their own proteolytic activity and substrate specificity (53). Omptins in *E. coli* mainly includes OmpT, which is encoded on chromosomes, and OmpP/ArlC, which are encoded on episomal plasmids (23, 38, 39). Most pathogenic *E. coli* strains (e.g., EPEC, EHEC, UPEC) only harbor OmpT on their chromosomes. However, we discovered that the APEC E058 strain (O2 serotype), a member of the ExPECs, possesses the p*ompT* on the *ColV* plasmid alongside the c*ompT* on the chromosome. Surprisingly, despite having only 76% amino acid sequence identity, both of these homologs are expressed on APEC E058’s outer membrane. Furthermore, the characterized active sites (including catalytic residues) and LPS-binding sites of cOmpT are conserved in pOmpT, with the exception of the two LPS binding sites. According to this study, APEC cOmpT and UPEC OmpT (from UPEC CFT073 and UPEC cystitis isolate 6) descended from the same ancestor, whereas pOmpT in APEC and ArlC in UPEC cystitis isolate 6 were homologs. OmpT can cleave protamine (35), which was confirmed by APEC E058. ArlC was discovered in the pathogenicity island on the plasmid of the AIEC NRG 857c strain and is involved in resistance to the human HDPs HD5, HBD2, and LL-37 (23). However, whether pOmpT or ArlC can resist protamine remains unclear. Here, the findings that pOmpT unable to cleave protamine fills the gap.

OmpT-like proteases exhibit variability in substrate specificity, indicating significant structural differences in the proteolytic active site enriched grooves between cOmpT and pOmpT despite their high amino acid sequence similarity (54). The only previously resolved structure of an *E. coli* OmpT-like subfamily protease was that of cOmpT, encoded on the chromosome. This structure featured a 10-chain antiparallel β-barrel, with its extracellular loop protruding above the lipid bilayer (24). Identified within this structural configuration were putative binding sites for LPS and the active sites (including the catalytic sites), located in the groove at the extracellular top of the vase-shaped β-barrel (24). Although software simulation results indicate that the overall structure of pOmpT closely resembles that of cOmpT, the true structure of pOmpT remains undiscovered. It has been found that omptins’ proteolytic activity and substrate specificity depend on residues within loops 1–5 (L1-L5), and mutations in these loops alter their activity (55). The analysis of cOmpT’s crystal structure confirms that the active sites are situated in the substrate-binding pocket region at the top of the extracellular loop (24). To investigate the resistance of protamine to pOmpT, we utilized the five extracellular loops of OmpT as entry points to examine the proteolytic activity and substrate specificity of pOmpT. The kinetics of protamine cleavage by loop-swap mutants indicated that L5 of cOmpT contributes to protamine cleavage activity. Furthermore, Asp^267^ and Ser^276^ in L5 of cOmpT were identified as the key residues that cleave protamine. Although the active sites and putative LPS-binding sites of cOmpT have been characterized, neither the 267th nor 276th residue is involved in these active sites. This result suggests that because of the characteristics of L5 and even its residues 267 and 276 of pOmpT, it loses the ability to resist protamine. Therefore, our findings are particularly important for supplementing critical information.

*Escherichia coli* OmpT was previously categorized as a serine protease, with residues Ser^99^ and His^212^ identified as typical active sites (56). Subsequent studies also classified OmpT as an aspartic protease (24), proposing a novel proteolytic mechanism involving a His^212^-Asp^210^ dyad and an Asp^83^-Asp^85^ pair that activates a putative nucleophilic water molecule, based on the crystal structure of *E. coli* OmpT (24). These active sites are fully conserved across the omptin family (24). This study investigates whether pOmpT operates via the same proteolytic mechanism as cOmpT and examines the impact of residues 267 and 276 on proteolytic activity. Sequence alignment analysis confirmed the conservation of these catalytic residues between the two homologs. Mutation of these four catalytic sites (Asp^83^, Asp^85^, Asp^210^ and His^212^) resulted in the complete abolition of the mutants’ proteolytic activity, demonstrating that neither residue 267 nor 276 interchange between cOmpT and pOmpT could restore the ability to cleave substrates, thereby indicating that these residues are not catalytic sites of OmpT. Earlier study supported the hypothesis that OmpT is a member of the serine protease family (57), since its proteolytic activity could be significantly inhibited by serine protease inhibitors such as diisopropylfluorophosphate (DFP) (27), phenylmethanesulfonyl fluoride (PMSF) (27), and aprotinin (49). Consistent with observations in cOmpT, the proteolytic activity of both wild-type pOmpT and its variant S267D/T276S were significantly inhibited by serine protease inhibitors (Aprotinin, PMSF, and Leupeptin), suggesting a shared catalytic mechanism between pOmpT and cOmpT. Subsequent analysis of the enzymatic activities of wild-type cOmpT, pOmpT, and their substitutions at residues 267 and 276 revealed a significantly lower proteolytic activity for pOmpT compared to cOmpT, aligning with pOmpT’s inability to cleave protamine. Substitution with either S276T or D267S/S276T significantly impaired the proteolytic activity of wild-type cOmpT; in contrast, replacement with S267D, T276S or S267D/T276S significantly enhanced the proteolytic activity of wild-type pOmpT. These results fully demonstrated that residues 267 and 276 of cOmpT and pOmpT are active sites that contribute to the proteolytic activity of both cOmpT and pOmpT, leading to different substrate cleavage specificities recognized by these two omptins. To elucidate the role of residues 267 and 276, the proteolytic kinetics of cOmpT, pOmpT, and their mutants involving these residues were examined using synthetic fluorescent peptides. Compared to the wild-type cOmpT, mutants displayed virtually unchanged catalytic constant (*K*_cat_), values but the *K*_m_ values were significantly increased: approximately 56% for the Asp^267^-to-Ser substitution [(16.7–10.7)/10.7], 344% for the Ser^276^-to-Thr substitution [(47.5–10.7)/10.7], and 281% for substitutions of both Asp^267^ and Ser^276^ [(40.8–10.7)/10.7]. Conversely, for pOmpT mutants relative to the wild-type pOmpT, the *K*_m_ values were reduced by about 58% (Ser^267^-to-Asp substitution, [(66.7–28.1)/66.7]), 76% (Thr^276^-to-Ser substitution, [(66.7–15.7)/66.7]) and 68% (both Ser^267^ and Thr^276^ substitutions, [(66.7–21.5)/66.7]). This suggests that residues 267 and 276 in both cOmpT and pOmpT are involved in substrate binding rather than catalysis, similar to Tyr^248^ in carboxypeptidase A, which is involved in substrate binding as indicated by a Tyr^248^-to-Phe substitution that maintained the same *K*_cat_ value while increasing its *K*_m_ value sixfold compared to the wild type (57). Thus, residues 267 and 276 in cOmpT and pOmpT likely serve as substrate binding sites, facilitating and promoting substrate cleavage by the catalytic center.

Protamine is a specific substrate for cOmpT but not for pOmpT; hence, we sought a specific substrate for pOmpT and investigated the mechanism underlying substrate cleavage specificity. ArlC in UPEC clinical isolates was reported to specifically cleave human AMP RNase 7 (23). However, it was observed in this study that both cOmpT and pOmpT from the APEC E058 strain could cleave human RNase 7. Substitutions of residues 267 and 276 between cOmpT and pOmpT also influenced their ability to cleave RNase 7, highlighting a unique feature of pOmpT from the APEC E058 strain: it does not cleave protamine but does cleave RNase 7, despite both being human AMPs. OmpT protease exhibits narrow cleavage specificity, preferring substrates at dibasic motifs (RR, RK, KK) (31, 32), a specificity determined by conserved residues Glu^27^ and Asp^208^ at the bottom of the deep S1 pocket and Asp^97^ in the shallower S1’ pocket (24). Despite the high conservation of these residues in pOmpT and cOmpT, molecular docking analysis suggests that residues 267 and 276 of cOmpT play a crucial role in substrate binding through interaction with arginine residues in protamine. This specificity variance between different omptins, driven by sequence variability in the outer loop (55), reveals differences in target substrate recognition by the 5-loop and its residues 267 and 276 of pOmpT, suggesting a structural specificity of pOmpT that prevents interaction with arginine residues in protamine, allowing for effective binding and cleavage of RNase 7 instead.

The evasion of APEC from host AMP killing effects remains a focal concern. OmpT, as a virulence factor, allows APEC to protect itself against the host. It is vital to understand whether the key sites determining substrate specificity influence APEC virulence. This study confirms that changes in the substrate-binding sites of cOmpT and pOmpT affect APEC infectivity, validating the hypothesis that OmpT-like proteases’ substrate specificity contributes to *E. coli* virulence. However, there was an unexplained outcome that substituting residue 267 on cOmpT with that of pOmpT in a pOmpT mutant (strain E058 c*ompT*_D267S_Δp*ompT*, 10^6.784^) was less pathogenic than the double mutant (strain E058Δc*ompT*Δp*ompT*, 10^5.499^), although the potential possibility of growth defect of this mutant had been ruled out *in vitro*. There were might some potential effects of residue 267 in cOmpT on other residues, even other virulence factors (*viz.* siderophores), leading to more attenuated virulence in the mutant. The findings were not only interesting, but also were worth making further study in future.

Given the WHO Reports in 2020 and 2021 highlighting the lack of effective treatments or preventions for bacterial infections due to antibiotic resistance, omptins could represent new targets for drug and vaccine development. Inhibiting or neutralizing these omptins is crucial in preventing septicemic bacterial infections (52, 53). Antimicrobial peptides, with their broad-spectrum antibacterial activity, low propensity for drug resistance development, and role in the innate immune system, have garnered significant interest (2, 58, 59). However, the natural composition of most antimicrobial peptides renders them susceptible to degradation by proteases like trypsin and pepsin in protease-rich physiological fluids (60). Therefore, antimicrobial peptides require modifications to resist enzymatic hydrolysis, enhancing their bactericidal efficacy for clinical application. Moreover, antimicrobial peptides also need to escape cleavage by bacterial omptins. For example, based on our findings, we can replace arginine at position 15 (R15) in protamine, which is readily bound to D267 in cOmpT, with the unnatural amino acid D-arginine, leading to escape from the recognition and binding of modified antimicrobial peptides by OmpT-like proteases. Since unnatural amino acids do not have protease recognition binding characteristics, their introduction into natural antimicrobial peptides can significantly reduce and even block protease degradation, ultimately improving antimicrobial activity (61).

In brief, we characterized the physiology of pOmpT, a newly discovered OmpT-like subfamily protease in pathogenic *E. coli* that causes intestinal and extraintestinal infections in both humans and poultry, and revealed its molecular mechanism involved in human AMP cleavage. Residues 267 and 276 were first characterized as substrate binding sites of omptins and play a critical role in the efficiency of AMP cleavage (62) and the pathogenicity of cOmpT and pOmpT. Importantly, both cOmpT and pOmpT of APEC, which were investigated in this study, are homologs of human ExPEC; in particular, cOmpT and pOmpT of avian origin confer APEC with the ability to cleave human AMPs, suggesting that APEC is a potential human pathogen (63). Our findings provide new insights for the development of antibacterial drugs that inhibit omptin activity.

## Data Availability

The datasets presented in this study can be found in online repositories. The names of the repository/repositories and accession number(s) can be found in the article/Supplementary material.

## Footnotes

^1^http://www.glschina.com/en/profile.htm

^2^https://www.rcsb.org

^3^https://swissmodel.expasy.org/

^4^https://alphafold.ebi.ac.uk

^5^http://autodock.scripps.edu
