# Supplementary material for: Protamine cleavage specificity of the avian pathogen Escherichia coli OmpT reveals two substrate-binding sites related to virulence
Source: Front Vet Sci. 2024 Sep 5;11:1410113. doi: 10.3389/fvets.2024.1410113 (PMC11410778; doi:10.3389/fvets.2024.1410113)

Supplementary Material


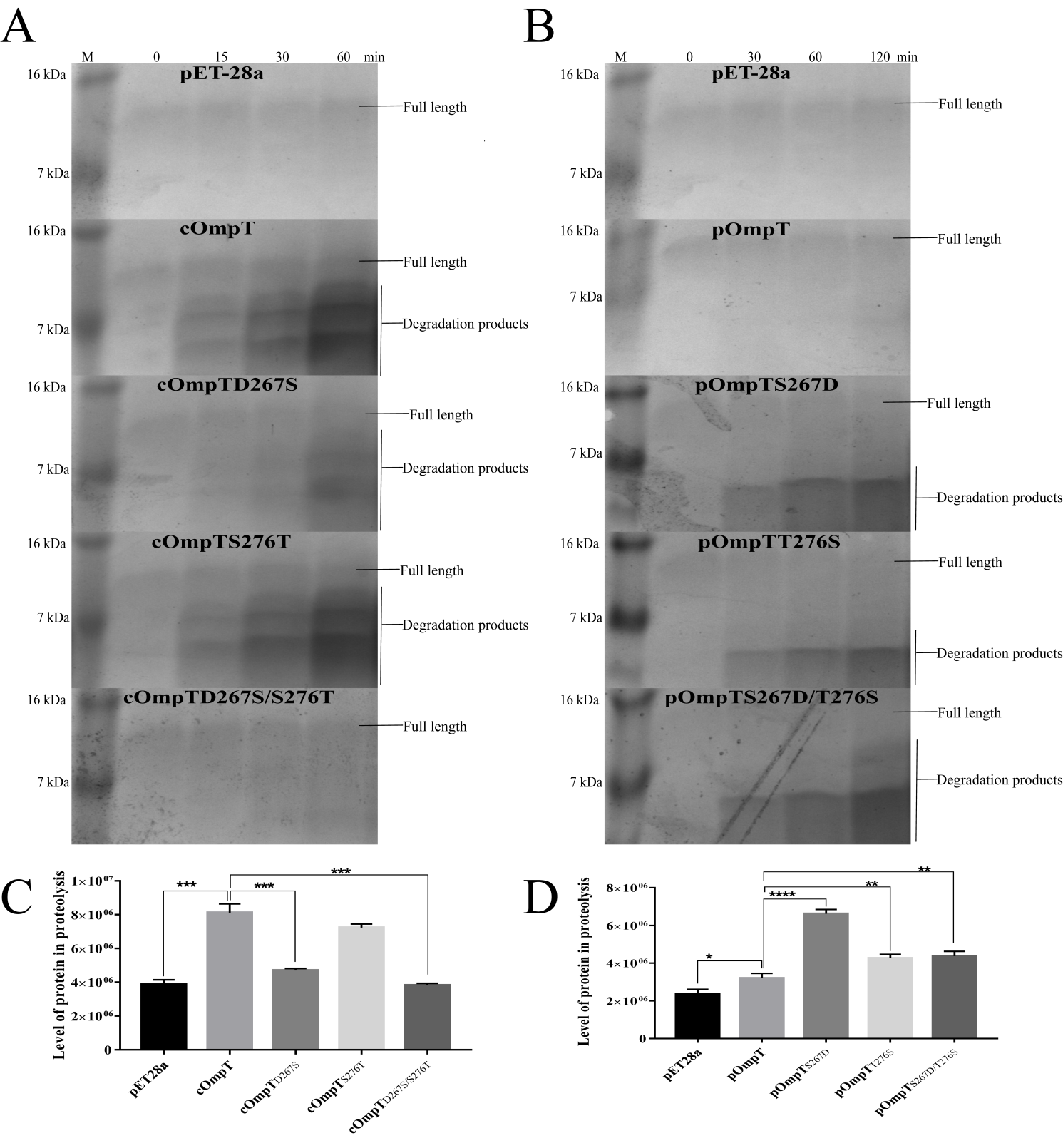


**Figure S1 Cleavage of protamine by cOmpT, pOmpT and their site-directed mutants.** **(A)** Protamine was incubated with *E*. *coli* BL21(DE3) expressing cOmpT (loops 1-5 of cOmpT) and its site-directed mutants (D267S and/or S276T) for the indicated times. **(B)** Protamine was incubated with *E. coli* BL21(DE3) expressing pOmpT(loops 1-5 of pOmpT) and its site-directed mutants(S267D and/or T276S) for the indicated times. **(C and D)** Quantitative analysis of the protamine products resolved by 16.5% Tris-Tricine SDS-PAGE and visualized with coomassie blue staining in **A** and **B** respectively. Statistical significance was determined using the *t*-test. Differences with *p*-values < 0.05 were considered as statistically significant. *: *p* < 0.05; **: *p* < 0.01; ***: *p* < 0.001; ****: *p* < 0.0001.

**Figure S2** **The conformation of protamine above the binding pocket of cOmpT, pOmpT and their mutants.** **(A and B)** cOmpT-protamine complex. **(C and D)** cOmpT_D267S/S276T_-protamine complex. **(E and F)** pOmpT_S267D/T276S_-protamine complex. Among them, **A**, **C** and **E** are their aerial views, respectively. cOmpT, pOmpT and their mutants were displayed by gray cartoon model. Protamine was displayed by green line model. In the stick model, orange represents residues on protamine, blue represents residues on OmpT, and purple represents catalytic residues on OmpT. Hydrogen bonds are represented by yellow dashed lines.


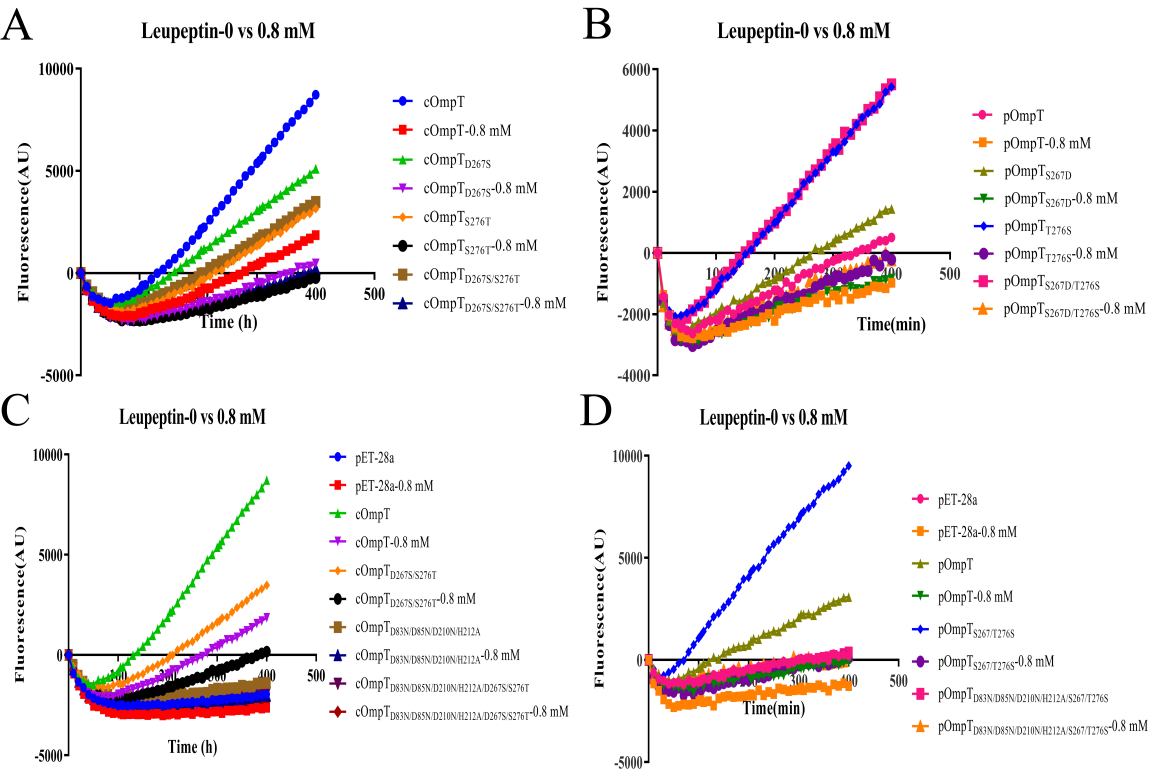


**Figure S3 Inhibition of enzyme activity of cOmpT, pOmpT and its site-directed mutants expressed in *E*. *coli* BL21 by leupeptin. (A, B, C and D)** FRET assays were performed with cOmpT, pOmpT and their site-directed mutants in PBS or in the presence of leupeptin (0.8 mM).


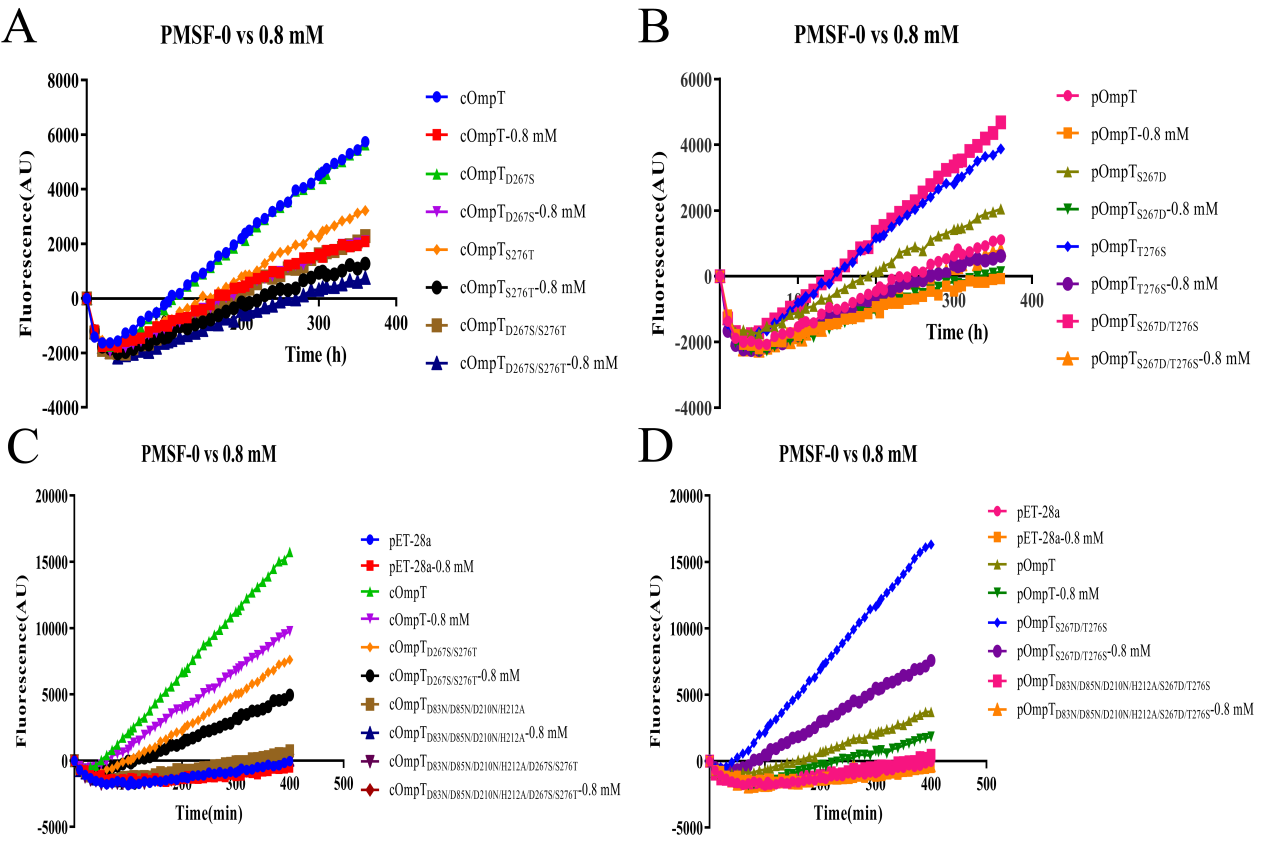


**Figure S4 Inhibition of enzyme activity of cOmpT, pOmpT and its site-directed mutants expressed in *E*. *coli* BL21 by PMSF. (A, B, C and D)** FRET assays were performed with cOmpT, pOmpT and their site-directed mutants in PBS or in the presence of PMSF (0.8 mM).

Table S1. Bacterial strains and plasmids used in this study.

| **Strains and plasmids** | **Characteristics** | **Source/**  **reference** |
| --- | --- | --- |
| **Strains** |  |  |
| E058 | Wild-type avian *E. coli* serotype O2 | (1) |
| E058Δc*ompT* | c*ompT* gene deficient strain | This study |
| E058Δp*ompT* | p*ompT* gene deficient strain | This study |
| E058Δc*ompT*Δp*ompT* | c*ompT* and p*ompT* gene deficient strain | This study |
| ReE058Δc*ompT-*cc*ompT* | Complementation of E058Δc*ompT* with c*ompT* gene with native c*ompT* putative promoter | This study |
| ReE058Δc*ompT-*pp*ompT* | Complementation of E058Δc*ompT* with p*ompT* gene with native p*ompT* putative promoter | This study |
| ReE058Δc*ompT-*p184 | Complementation of E058Δc*ompT* with plasmids pACYC 184 | This study |
| E058c*ompT*_c1-4p5_Δp*ompT* | The loops 1-4 (L1-L4) of c*ompT* gene and the loop 5 (L5) of p*ompT* gene were chimerized and replaced the c*ompT* gene, while the p*ompT* gene was mutated in wild-type strain E058 | This study |
| E058c*ompT*_D267S_Δp*ompT* | Aspartic acid (Asp, D) at position 267 in c*ompT* gene was replaced with serine (Ser, S), while the p*ompT* gene was mutated in wild-type strain E058 | This study |
| E058c*ompT*_S276T_Δp*ompT* | Serine (Ser, S) at position 276 in c*ompT* gene was replaced with threonine (Thr, T), while the p*ompT* gene was mutated in wild-type strain E058 | This study |
| E058c*ompT*_D267S/S276T_Δp*ompT* | Both D at position 267 and S at position 276 in c*ompT* gene were replaced with S and T, respectively, while the p*ompT* gene was mutated in wild-type strain E058 | This study |
| E058c*ompT*_D83N/D85N/D210N/H212A_Δp*ompT* | Each D at positions 83, 85, 210 in c*ompT* gene was replaced with asparagine (Asn, N), respectively. Further, histidine (His, H) at position 212 in *compT* gene was replaced with alanine (Ala, A), while the p*ompT* gene was mutated in wild-type strain E058 | This study |
| E058c*ompT*_D83N/D85N/D210N/H212A/D267S/S276T_Δp*ompT* | Each D at positions 83, 85, 210 in c*ompT* gene was replaced with N, respectively. In addition, in *compT* gene, H at position 212, D at position 267 and S at position 276 were replaced with A, S, and T, respectively, while the p*ompT* gene was mutated in wild-type strain E058 | This study |
| E058p*ompT*_p1-4c5_Δc*ompT* | The L1-L4 of p*ompT* gene and the L5 of c*ompT* gene were chimerized and replaced the p*ompT* gene, while the c*ompT* gene was mutated in wild-type strain E058 | This study |
| E058p*ompT*_S267D_Δc*ompT* | S at position 267 in p*ompT* gene was replaced with D, while the c*ompT* gene was mutated in wild-type strain E058 | This study |
| E058p*ompT*_T276S_Δc*ompT* | T at position 276 in p*ompT* gene was replaced with S, while the c*ompT* gene was mutated in wild-type strain E058 | This study |
| E058p*ompT*_S267D/T276S_Δc*ompT* | S at position 267 and T at position 276 in p*ompT* gene were replaced with D and S, respectively, while the c*ompT* gene was mutated in wild-type strain E058 | This study |
| E058p*ompT*_D83N/D85N/D210N/H212A/S267D/T276S_Δc*ompT* | Each D at positions 83, 85, 210 in p*ompT* gene was replaced with N, respectively. In addition, in p*ompT* gene, H at position 212, S at position 267, and T at position 276 were replaced with A, D, and S, respectively, while the c*ompT* gene was mutated in wild-type strain E058 | This study |
| pET28a/BL21 | The vector pET-28a was transformed into BL21 | This study |
| pET-cOmpT/BL21 | The c*ompT* genes of the whole open reading frame (ORF) (with signal peptide and loops 1-5 (L1-L5)) cloned into pET-28a was transformed into BL21 | This study |
| pET-pOmpT/BL21 | The p*ompT* genes of the whole ORF (with signal peptide and loops 1-5 (L1-L5)) cloned into pET-28a was transformed into BL21 | This study |
| pET-c1-3p4-5/BL21 | The signal peptide and loops 1-3 (L1-L3) of c*ompT* gene and the loops 4-5 (L4-L5) of p*ompT* gene were chimerized and cloned into pET-28a, and transformed into BL21 | This study |
| pET-p1-3c4-5/BL21 | The signal peptide and L1-L3 of p*ompT* gene and the L4-L5 of c*ompT* gene were chimerized and cloned into pET-28a, and transformed into BL21 | This study |
| pET-c1-4p5/BL21 | The signal peptide and L1-L4 of c*ompT* gene and the L5 of p*ompT* gene were chimerized and cloned into pET-28a, and transformed into BL21 | This study |
| pET-p1-4c5/BL21 | The signal peptide and L1-L4 of p*ompT* gene and the L5 of c*ompT* gene were chimerized and cloned into pET-28a, and transformed into BL21 | This study |
| pET-c1-3p4c5/BL21 | The signal peptide, L1-L3 and L5 of c*ompT* gene and the L4 of p*ompT* gene were chimerized and cloned into pET-28a,and transformed into BL21 | This study |
| pET-p1-3c4p5/BL21 | The signal peptide, L1-L3 and L5 of p*ompT* gene and the L4 of c*ompT* gene were chimerized and cloned into pET-28a,and transformed into BL21 | This study |
| pET-cOmpT_D267S_/BL21 | The c*ompT* gene of the whole ORF with D at position 267 substituted by S was cloned into pET-28a, and transformed into BL21 | This study |
| pET-pOmpT_S267D_/BL21 | The p*ompT* gene of the whole ORF with S at position 267 substituted by D was cloned into pET-28a, and transformed into BL21 | This study |
| pET-cOmpT_S276T_/BL21 | The c*ompT* gene of the whole ORF with S at position 276 substituted by T was cloned into pET-28a, and transformed into BL21 | This study |
| pET-pOmpT_T276S_/BL21 | The p*ompT* gene of the whole ORF with T at position 276 substituted by S was cloned into pET-28a, and transformed into BL21 | This study |
| pET-cOmpT_D267S/S276T_/BL21 | The c*ompT* gene of the whole ORF with D at position 267 and S at position 276 substituted by S and T respectively was cloned into pET-28a, and transformed into BL21 | This study |
| pET-pOmpT_S267D/T276S_/BL21 | The p*ompT* gene of the whole ORF with S at position 267 and T at position 276 substituted by D and S respectively was cloned into pET-28a, and transformed into BL21 | This study |
| pET-cOmpT_D83N/D85N/D210N/H212A_/BL21 | The c*ompT* gene of the whole ORF with each D at positions 83, 85, 210 substituted by N respectively and H at position 212 instead by A was cloned into pET-28a, and transformed into BL21 | This study |
| pET-cOmpT_D83N/D85N/D210N/H212A/D267S/S276T_/BL21 | The c*ompT* gene of the whole ORF with each D at positions 83, 85, 210 substituted by N respectively, further, H at position 212, D at position 267 and S at position 276 replaced with A, S and T respectively, was cloned into pET-28a, and transformed into BL21 | This study |
| pET-pOmpT_D83N/D85N/D210N/H212A/S267D/T276S_/BL21 | The p*ompT* gene of the whole ORF with each D at positions 83, 85, 210 substituted by N respectively, further, H at position 212, S at position 267 and T at position 276 replaced with A, D, and S respectively, was cloned into pET-28a, and transformed into BL21 | This study |
| pDEST17-RNase 7/BL21(AI) | The *RNase 7* gene of the whole ORF without signal peptide, but with proline (Pro, P) at position 3 substituted by A and tyrosine (Tyr, Y) at position 16 by H was cloned into pDEST17, and transformed into BL21(AI) | This study |
| BL21(DE3) *E. coli* | F- *ompT hsdSB*(r_B_^-^ m_B_^-^) *gal* *dcm*(DE3) (deficient in the outer membrane protease, OmpT and lon protease) | Stored in our laboratory |
| BL21(AI) *E. coli* | F- *ompT hsdSB*(r_B_^-^ m_B_^-^) *gal* *dcm araB*::*T7RNAP-tetA* | Weidi biotechnology |
| **Plasmids** |  |  |
| pACYC 184 | Cloning Vector, *tet* and *cat* resistant gene | Stored in our laboratory |
| pET-28a | Expression vector, kanamycin resistance cassette | Stored in our laboratory |
| pDEST17 | Expression vector, ampicillin resistance cassette | Youbio biotechnolo-gy |

Table S2. Primers used in this study.

| **Primers and probes** | **Primer sequence 5’–3’** | **Application** |
| --- | --- | --- |
| **Primers** |  |  |
| c*ompT*-*cat*-F | ATTCCCCGGGGCTATACAATACCACCGGGGAGAAAATCTATTTAACGTTGTTGTGTAGGCTGGAGCTGCT | Construction of mutant strains E058Δc*ompT* and E058Δc*ompT*Δp*ompT* |
| c*ompT*-*cat*-R | ATATAAAAAATACATACTCAATCATTAAAACGATTGAATGGAGACCTTTTATGGGAATTAGCCATGGTCC |  |
| p*ompT*-*cat*-F | TAAAAAAATATTTATCAAAAACAAACGAGTGGAATGGAGTTAACTTGTCTATGGGAATTAGCCATGGTCC | Construction of mutant strains E058Δp*ompT* and E058Δc*ompT*Δp*ompT* |
| p*ompT*-*cat*-R | TTATCATATTTAAAGTGTCATGAAAAAGTTAAAAACAGGCATGCTTCAGGTTGTGTAGGCTGGAGCTGCT |  |
| Recc*ompT*-F | AATTATCCGCGGCACGACTTAGAAACTCCAGGA *Sac*II | Construction of plasmid p-cc*ompT* |
| Rec*ompT*-R | CGCGCGAGTACTTTAAAAGGTGTACTTAAGAAC *Sca*I |  |
| Repp*ompT*-F | AATTATCCGCGGCGGCCACATTGTAAACAAAC *Sac*II | Construction of plasmid p-pp*ompT* |
| Rep*ompT*-R | CGCGCGAGTACTTTAGAAATAATACTTCAGACC *Sca* I |  |
| c-F | ATAACCATGGTTATGCGGGCGAAACTTCT *Nco*I | Construction of plasmid pET-cOmpT |
| c-R | CGCGCTCGAGTTAAAAGGTGTACTTAAGAC *Xho*I |  |
| p-F | GCGGCCATGGGCATGTACTTAAAGATTCTTGC *Nco*I | Construction of plasmid pET-pOmpT |
| p-R | CGCGCTCGAGTTACAAATAATACTTCAGACCA *Xho*I |  |
| c3:p4-Rm | CCCGAACTCAAAGCTTTCATAACGATAACT | Construction of plasmid pET-c1-3p4-5 |
| c3:p4-Fm | AGTTATCGTTATGAAAGCTTTGAGTTCGGG |  |
| p3:c4-Rm | GCCACCTAGCTCAAAGCTATCATACCGATA | Construction of plasmid pET-p1-3c4-5 |
| p3:c4-Fm | TATCGGTATGATAGCTTTGAGCTAGGTGGC |  |
| c4:p5-Rm | AAACTTTAGCTGCAGGTGTTACGTAA | Construction of plasmids pET-c1-4p5 and pET-p1-3c4p5 |
| c4:p5-Fm | TTACGTAACACCTGCAGCTAAAGTTT |  |
| p4:c5-Rm | AACCTTTGCGTTAGGTGTGATGTAA | Construction of plasmids pET-p1-4c5 and pET-c1-3p4c5 |
| p4:c5-Fm | 0TTACATCACACCTAACGCAAAGGTT |  |
| c267-Rm | CATTGTGGGAATAAAGCGAAG | Construction of plasmids pET-cOmpT_D267S_ and pET-cOmpT_D267S/S276T_ |
| c267-Fm | CTTCGCTTTATUCCCACAATG |  |
| p267-Rm | CAAGTTTCTGTCGTACAGAGA | Construction of plasmids pET-pOmpT_S267D_ and pET-pOmpT_S267D/T276S_ |
| p267-Fm | TCTCTGTACGACAGAAACTTG |  |
| c276-Rm | CACCATTTTTGGTGTAGTCAG | Construction of plasmids pET-cOmpT_S276T_ and pET-cOmpT_D267S/S276T_ |
| c276-Fm | CTGACTACACCAAAAATGGTG |  |
| p276-Rm | CACCATTTTTGCTGTGATCAG | Construction of plasmids pET-pOmpT_T276S_ and pET-pOmpT_S267D/T276S_ |
| p276-Fm | CTGATCACAGCAAAAATGGTG |  |
| c83/85-Rm | CCATCCAGTTCTGATTGACCA | Construction of plasmids pET-cOmpT_D83N/D85N/D210N/H212A_ and pET-cOmpT_D83N/D85N/D210N/H212A/D267S/S276T_ |
| c83/85-Fm | TGGTCAATCAGAACTGGATGG |  |
| c210/212-Rm | CTGGGTCATAGGCCTCATTGT |  |
| c210/212-Fm | ACAATGAGGCCTATGACCCAG |  |
| p83/85-Rm | AGCCAGTTCCTGTTCACCATA | Construction of plasmid pET-pOmpT_D83N/D85N/D210N/H212A/S267D/T276S_ |
| p83/85-Fm | TATGGTGAACAGGAACTGGCT |  |
| p210/212-Rm | CTCAGGGTTGTAAGCCTCGTTA |  |
| p210/212-Fm | GGATAATAACGAGGCTTACAACCCTGAG |  |
| pTarget F-N20-FRT-F | GTCCTAGGTATAATACTAGTTCCTATACTTTCTAGAGAATGTTTTAGAGCTAGAAATAG | Construction of plasmid pTarget F-FRT |
| pTarget F-N20-c*ompT*-F | GTCCTAGGTATAATACTAGTAAAAGAGCTGATCGCAATAGGTTTTAGAGCTAGAAATAGC | Construction of plasmid pTarget F-c*ompT* |
| pTarget F-N20-p*ompT*-F | GTCCTAGGTATAATACTAGTGCCAGTGCAGCAAACGCTACGTTTTAGAGCTAGAAATAGC | Construction of plasmid pTarget F-p*ompT* |
| Target F-R | ACTAGTATTATACCTAGGACTGAG | Construction of plasmids pTarget F-FRT, pTarget F-c*ompT* and pTarget F-p*ompT* |
| cCRISPR-c*ompT*-F | ATTCCCCGGGGCTATACAATACCACCGGGGAGAAAATCTATTTAACGTTGTTAAAAGGTGTACTTAAGAC | Construction of donor DNA d-cFRT, d-ccOmpT_c1-4p5_, d-ccOmpT_D267S_, d-ccOmpT_S276T_, d-ccOmpT_D267S/S276T_, d-ccOmpT_D83N/D85N/D210N/H212A_ and d-ccOmpT_D83N/D85N/D210N/H212A/D267S/S276T_ |
| cCRISPR-c*ompT*-R | GATATAAAAAATACATACTCAATCATTAAAACGATTGAATGGAGACCTTTTATGCGGGCGAAACTTCTGGG |  |
| cCRISPR-pL5-F | ATTCCCCGGGGCTATACAATACCACCGGGGAGAAAATCTATTTAACGTTGTTAAAAATAATACTTCAGAC |  |
| pCRISPR-p*ompT*-F | TAAAAAAATATTTATCAAAAACAAACGAGTGGAATGGAGTTAACTTGTCT  ATGTACTTAAAGATTCTTGC | Construction of donor DNA d-pFRT, d-ppOmpT_p1-4c5_, d-ppOmpT_S267D_, d-ppOmpT_T276S_, d-ppOmpT_S267D/T276S_, and d-ppOmpT_D83N/D85N/D210N/H212A/S267D/T276S_ |
| pCRISPR-p*ompT*-R | TTATCATATTTAAAGTGTCATGAAAAAGTTAAAAACAGGCATGCTTCAGGTTAAAAATAATACTTCAGAC |  |
| pCRISPR-cL5-R | TTATCATATTTAAAGTGTCATGAAAAAGTTAAAAACAGGCATGCTTCAGGTTAAAAGGTGTACTTAAGAC |  |
| c*ompT*(WJD)-F | AAATTCCCCGGGGCTATA | PCR identification of site-directed mutagenesis’ insertion and deletion of c*ompT*/p*ompT* gene |
| c*ompT*(WJD)-R | ACGATTGAATGGAGACCT |  |
| p*ompT*(WJD)-F | AGTTGACTACTACGAGT |  |
| p*ompT*(WJD)-R | AGTGCTGGCCTTTTGTT |  |
| His-F | ACGCATATGTCGTACTACCATCACCATCACCATCACCTCGAATCAACAAGTTTGTACAA | Construction of plasmid pDEST17-RNase 7 |
| His-R | GGCTTCTTGTCGTCGTCGTCGGTACCAGCTTTTTTGTACAAACTTGTTGATTCGAGGTG |  |
| RK-F | AGCTGGTACCGACGACGACGACAAGAAGCCCAAGGGCATGACCTC |  |
| P3A-Y16H-F | ACGGGGCAGTGTCCCTGACCATGTGTAAGCTCACCTCAGGGAAGCACCCGAACTGCAGG |  |
| P3A-Y16H-R | GTTCGGGTGCTTCCCTGAGGTGAGCTTACACATGGTCAGGGACACTGCCCCGTGGCTCT |  |
| RK-R | TATGCGGCCGCCTAAAGGACTCTGTCCAAGTGTACAGG |  |

**Reference**

1. Gao S, Liu X, Zhang R, Jiao X, Wen Q, Wu C, et al. The Isolation and Identification of Pathogenic *Escherichia coli* Isolates of Chicken Origin from Some Regions in China. *Acta Veterinaria et Zootechnica Sinica* (1999) 30:164-71.

**The original blot and gels of Cropped images shown in figures**

Figure 1A


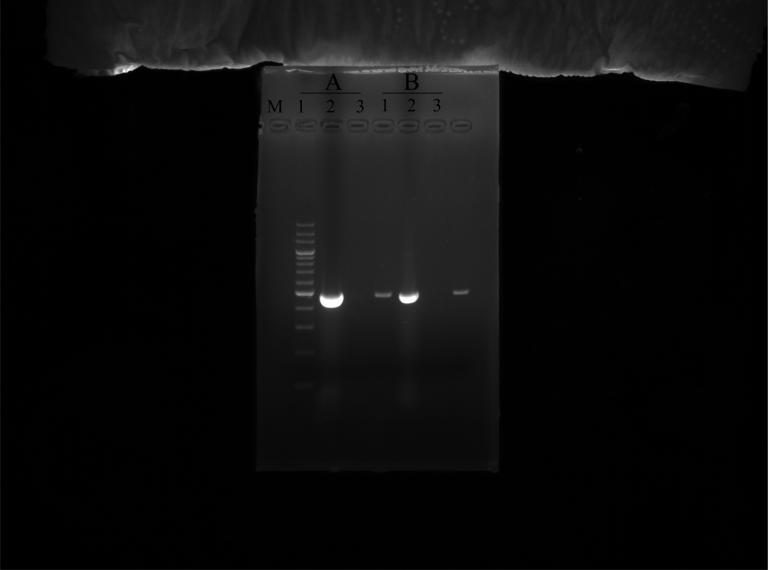


Figure 1C


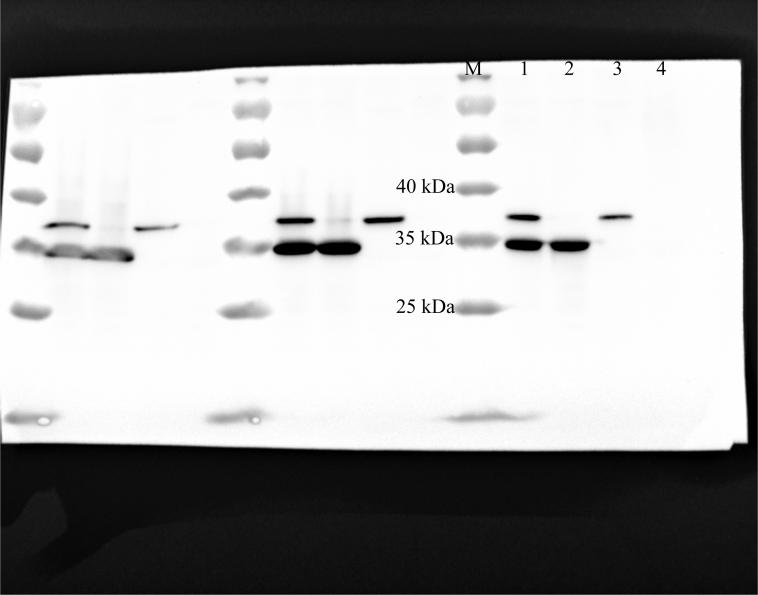


Figure 6


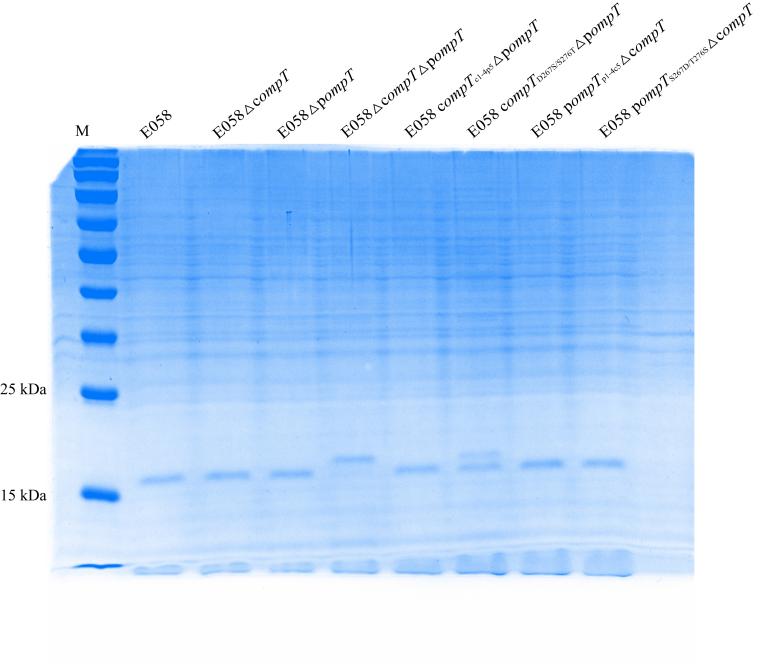


Figure S1A pET-28a


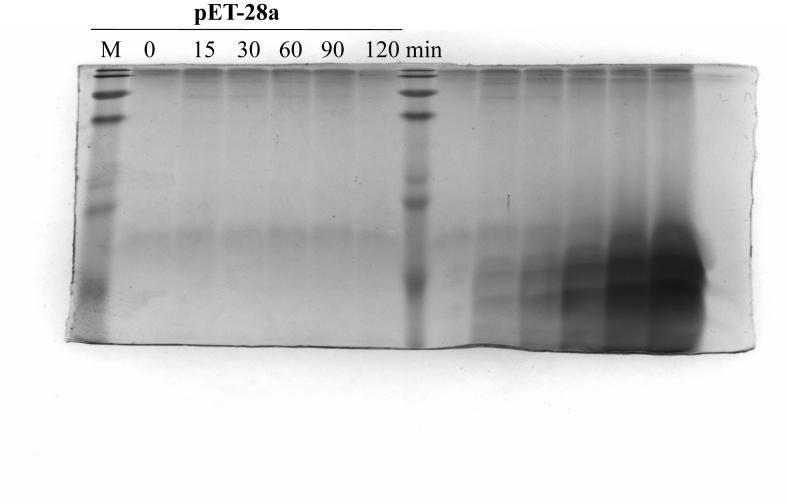


Figure S1A cOmpT


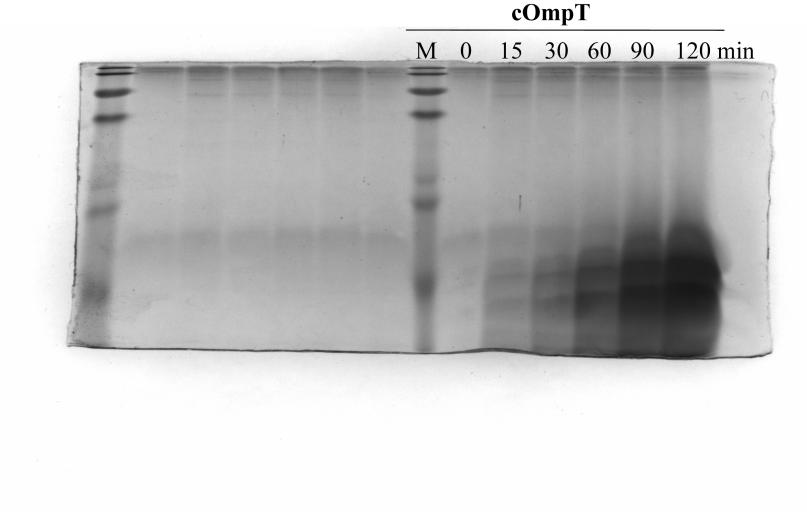


Figure S1A cOmpT_D267S_


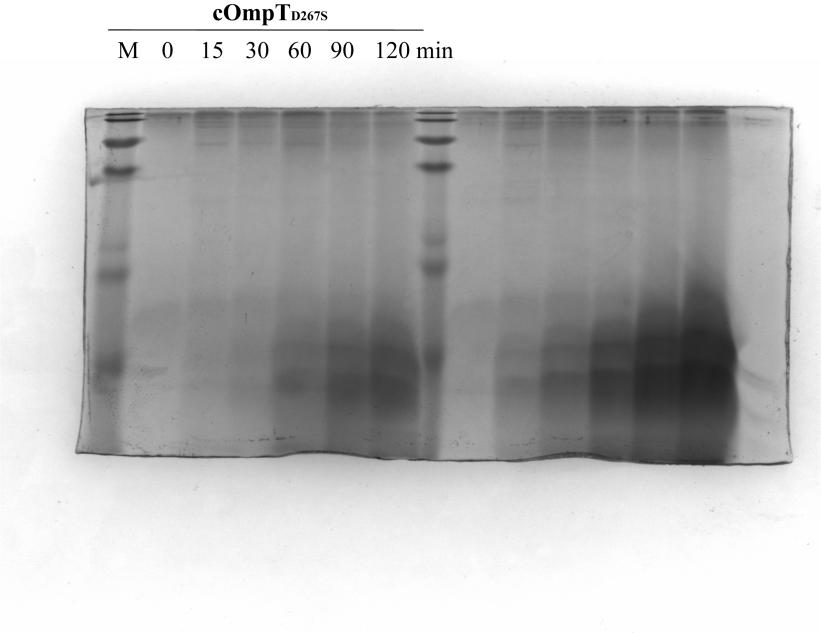


Figure S1A cOmpT_S276T_


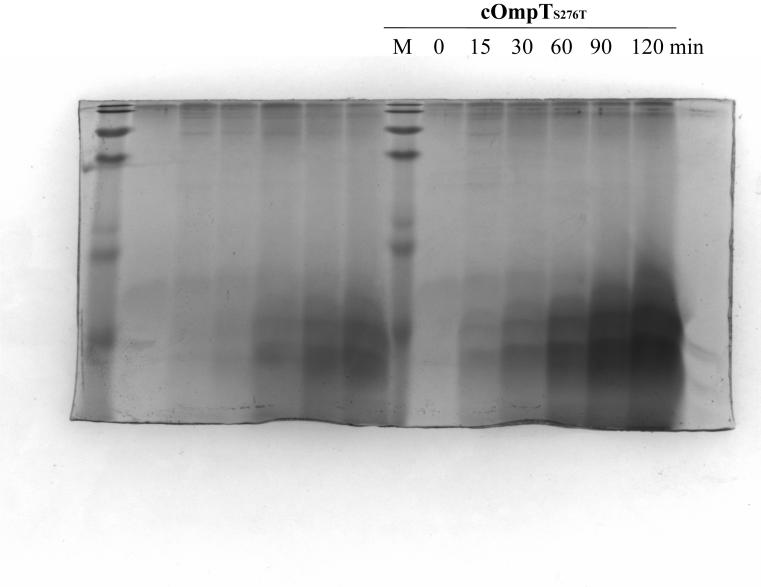


Figure S1A cOmpT_D267S/S276T_


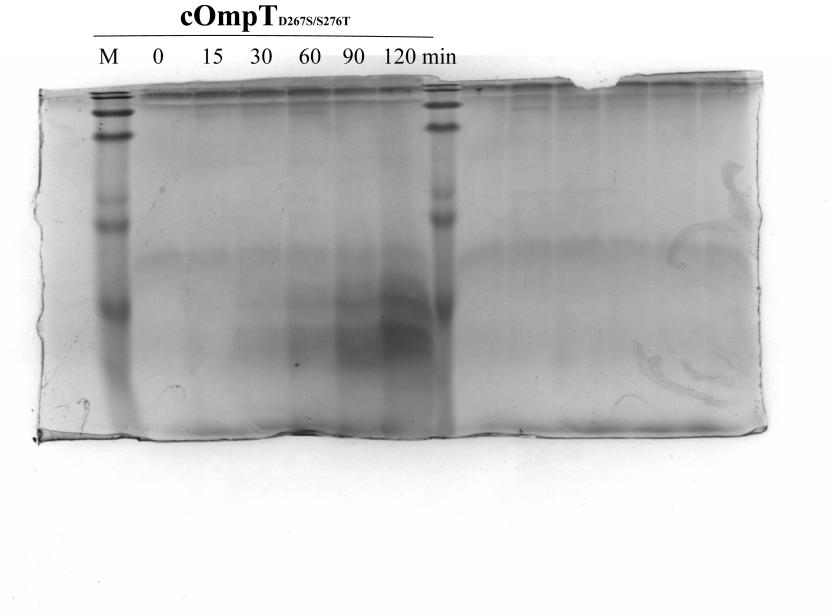


Figure S1A pET-28a


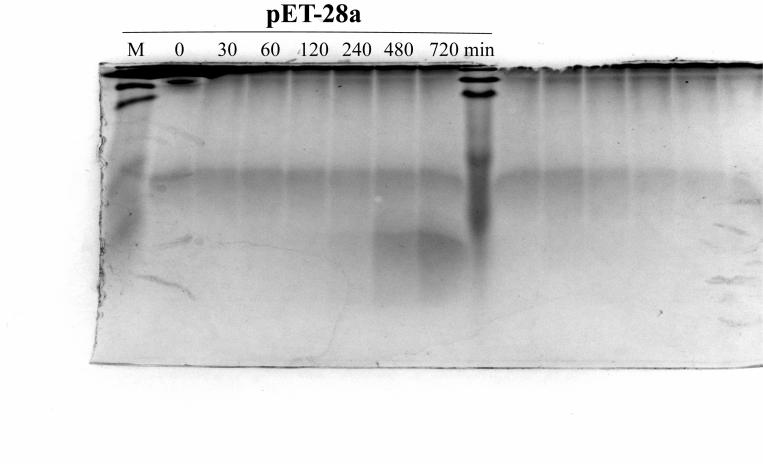


Figure S1B pOmpT


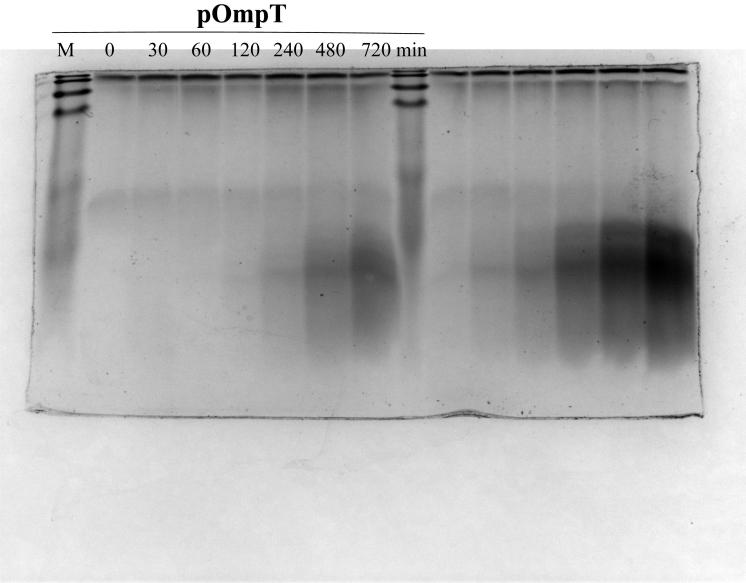


Figure S1B pOmpT_S267D_


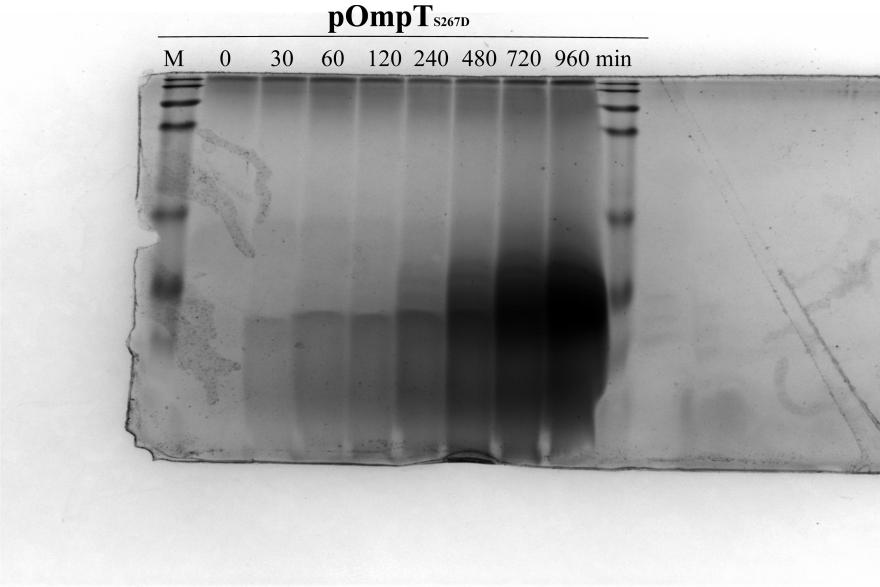


Figure S1B pOmpT_T276S_


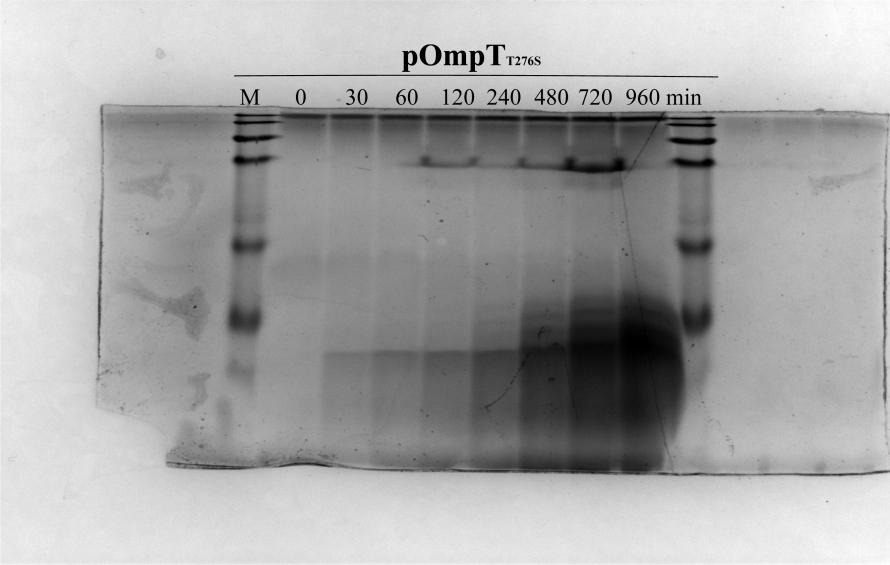


Figure S1B pOmpT_S267D/T276S_


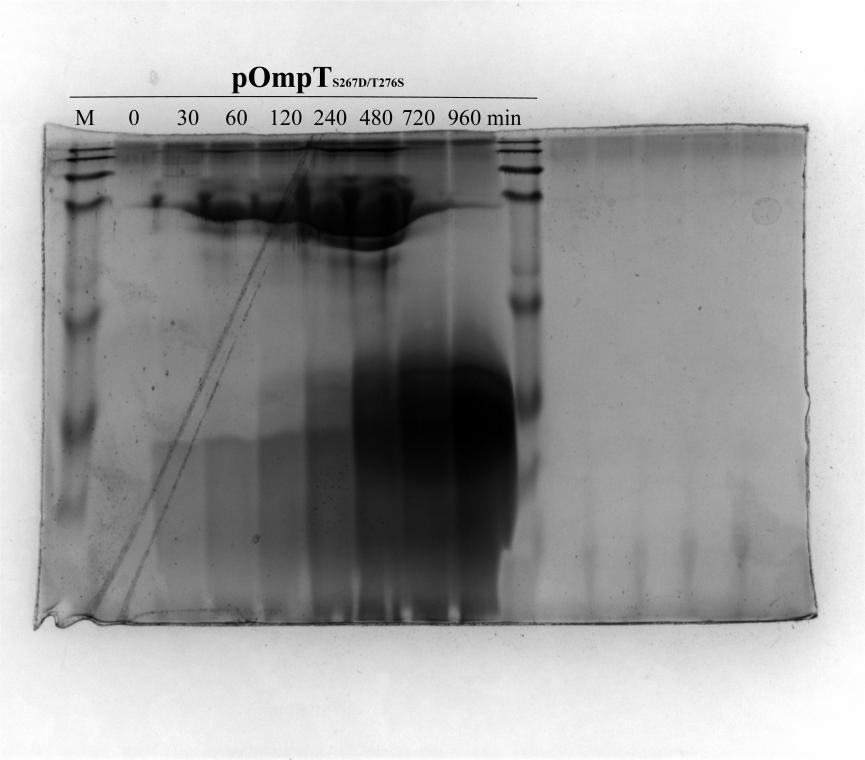

Supplement: Supplementary file 1 [file Data_Sheet_1.docx]
